# Using the Pleiotropic Characteristics of Curcumin to Validate the Potential Application of a Novel Gene Expression Screening Platform

**DOI:** 10.3390/nu11061397

**Published:** 2019-06-21

**Authors:** Se-Chun Liao, Hsiu-Wen Hsu, Kun-Lin Chuang, Zi-Yi Huang, Kuan-Ting Lin, Wei-Hsiang Hsu, Kai-Hsun Chang, Chi-Yin F. Huang, Chun-Li Su

**Affiliations:** 1 Institute of Clinical Medicine, National Yang-Ming University, Taipei 112, Taiwan; susanliao@everestpharm.com; 2 Department of Human Development and Family Studies, National Taiwan Normal University, Taipei 106, Taiwan; sw760911@hotmail.com (H.-W.H.); e0208ric@hotmail.com (K.-H.C.); 3 Institute of Biopharmaceutical Sciences, National Yang-Ming University, Taipei 112, Taiwan; jason101024004@gmail.com (K.-L.C.); star34179616@gmail.com (Z.-Y.H.); rabbitjim5@hotmail.com (W.-H.H.); 4 Cold Spring Harbor Laboratory, Cold Spring Harbor, New York, NY 11724, USA; woodydon777@gmail.com; 5 Department of Biochemistry, College of Medicine, Kaohsiung Medical University, Kaohsiung 807, Taiwan; 6 Graduate Program of Nutrition Science, School of Life Science, National Taiwan Normal University, Taipei 106, Taiwan

**Keywords:** curcumin, NF-κB, Connectivity map, library of integrated network-based cellular signatures, C-Map and LINCS unified environment, ConsensusPathDB

## Abstract

Curcumin is a polyphenol derived from *curcumin longa* that exhibits anticancer and anti-inflammatory properties. The consumption of foods at supernutritional levels to obtain health benefits may paradoxically result in negative health outcomes. In the present study, multiple targeting characteristics of curcumin were analyzed using our gene expression screening system, which utilized the gene expression signatures of curcumin from human hepatocellular carcinoma and colorectal cancer cells to query gene expression databases and effectively identify the molecular actions of curcumin. In agreement with prediction, curcumin inhibited NF-κB and Aurora-A, and induced G2/M arrest and apoptosis. Curcumin-suppressed NF-κB was identified through inhibition of PLCG1, PIK3R1, and MALT1 in the CD4-T-cell-receptor-signaling NF-κB cascade pathway. The results suggest that our novel gene expression screening platform is an effective method of rapidly identifying unknown biological functions and side effects of compounds with potential nutraceutical benefits.

## 1. Introduction

Curcumin (diferuloylmethane) is a bioactive and pharmacologically efficient component isolated from the Indian spice turmeric [1]. Curcumin exhibits diverse pharmacologic activities, including antioxidant, anti-inflammatory, anticancer, and immune-regulatory effects that can prevent diabetes and protect the liver as well as the nervous and cardiovascular systems [2].

The Connectivity map (C-Map) is a database that enables the identification of connections among genes, drugs, and diseases through gene expression signatures [3]. The C-Map contains more than 7000 gene expression profiles representing 1309 compounds. By querying the database, the level of similarity between gene expression signatures affected by a compound of interest can be calculated. Moreover, by exploring the relationship between genes and disease, one or more drugs that can potentially reverse the current gene signature may be identified, and such identification could benefit cellular physiology.

Another platform that we used to process the gene signature is called C-Map and library of integrated network-based cellular signatures (LINCS) unified environment (CLUE) [4], a program launched by the United States’ National Institutes of Health that is the latest version of the LINCS database. The purpose of the CLUE database is to facilitate user engagement with highly dimensional data [5]. Analysis results are not merely presented as a list of similar compounds; they also show the perturbagen class (PCL). Compound of PCLs are identified by first grouping compounds that have the same biological functions as those reported in the literature. Subsequently, the L1000 data are analyzed for these groupings to determine whether the members yield similar gene expression signatures in order to confirm their shared activity.

ConsensusPathDB (CPDB) is a database that integrates various intracellular information concerning interactions among genes, RNA, proteins, protein complexes, and metabolites to assemble a relatively comprehensive and unbiased cellular biology signal result [6]. The current CPDB data set contains metabolic and signaling reactions, physical protein interactions, genetic interactions, gene regulatory interactions, and drug–target interactions in humans, mice, and yeast. Analysis data are collected from 30 public resources and integrated into a seamless network. In addition, interactions among matching primary participants (i.e., substrates and products in cases of biochemical reactions, interactors in cases of protein interactions, and regulated genes in cases of gene regulatory interactions) are mapped and grouped according to similarity [7].

The present study applied a gene expression screening system that utilizes the unique profiling platform of the C-Map, CLUE, and CPDB to analyze multiple known characteristics of curcumin as positive controls and to extend this technique for effective identification of novel molecular actions of curcumin.

## 2. Materials and Methods

### 2.1. Materials

All of the chemicals used in this study were obtained from Sigma (St. Louis, MO, USA) unless otherwise indicated. Anti-Aurora-A, anti-phospho-Aurora-A (Thr288), anti-caspase 3, and anti-phospho-IκBα (Ser32-36) antibodies were obtained from Cell Signaling Technology (Danvers, MA, USA). Anti-IκBα antibody was obtained from Abcam (Cambridge Science Park, Cambridge, UK). Anti-PLCG1, anti-PIK3R1, and anti-GAPDH antibodies were obtained from Abnova Corporation (Taipei, Taiwan). Anti-NF-κB p65 and anti-MALT1 antibodies and anti-rabbit and anti-mouse IgG-conjugated horseradish peroxidase secondary antibodies were obtained from Santa Cruz Biotechnology (Santa Cruz, CA, USA).

### 2.2. Determination of Gene Expression Profiles of Curcumin Using the L1000 Microarray

Human hepatocellular carcinoma HepG2 and human colorectal cancer HT29 cell lines (American Type Culture Collection [ATCC], Rockville, MD, USA) were treated in triplicate with 20 μM of curcumin dissolved in dimethyl sulfoxide. These samples were then submitted to Genometry, Inc. (Cambridge, MA, USA) for L1000 microarray profiling. The microarray gene expression profiling results were classified according to the up- and down-regulated gene signatures, which were subsequently used to query the C-Map [3] and CLUE [4] databases for analysis of the gene expression of curcumin. Additional details are listed in Supplementary materials.

### 2.3. Cell Culture

Human hepatocellular carcinoma HepG2 (ATCC), Huh7 (provided by Dr. Zhong-Zhe Lin, National Taiwan University Hospital, Taipei, Taiwan), Huh7R (Dr. Zhong-Zhe Lin), and colorectal cancer HT29 (ATCC) cells were maintained in complete Dulbecco’s Modified Eagle Medium (GIBCO BRL, Gaithersburg, MD, USA) or Roswell Park Memorial Institute 1640 Medium (no HEPES; GIBCO BRL) containing 10% fetal bovine serum (Biological Industries Ltd., Kibbutz Beit Haemek, Israel; Invitrogen, Carlsbad, CA, USA) and placed in an incubator at 37 °C with a humidified atmosphere of 5% CO_2_. The curcumin dissolved in dimethyl sulfoxide was stored at −20 °C. Before the experiments, the curcumin stock was diluted to the final indicated concentrations with complete Dulbecco’s Modified Eagle Medium; control cells were cultured in a medium containing an equal amount of dimethyl sulfoxide without curcumin.

### 2.4. Immunoblotting

Whole cell lysates and nuclei-enriched fractions underwent Western blot analysis [8]. Protein contents determined by a protein assay kit (Bio-Rad Laboratories, Hercules, CA, USA) were resolved using 8%–12% SDS-PAGE and subsequently transferred to polyvinylidene fluoride membranes (Millipore Corporation, Billerica, MA, USA). After hybridization with primary and secondary antibodies, the signaling was examined using a Biospectrum Imaging System (Universal Hood II, Bio-Rad Laboratories, Hercules, CA, USA).

### 2.5. Flow Cytometric Analysis

Because apoptotic signaling cascades lead to DNA fragmentation and apoptotic body formation [9], apoptotic cells with reduced DNA content (hypodiploid, <2N) produce a peak at the sub-G1 position [8]. Therefore, the percentages of cells at the sub-G1 phase represent the proportion of apoptotic cells. To determine the cell cycle distribution and induction of apoptosis in response to curcumin, the fluorescent dye propidium iodide, which stains nucleic acids, was added to the cells. After treatment, the stained cells were examined using a flow cytometer (Becton Dickson, Lexington, KY, USA) [10]. The results were analyzed using FlowJo (Tree Star Inc., Ashland, OR, USA).

### 2.6. Determination of Cell Population Growth

The population growth of HT29 was evaluated using the modified colorimetric assay. The Dulbecco’s Modified Eagle Medium containing curcumin was removed after treatment to prevent color interference. Next, 3-[4,5-Dimethylthiazol-2-yl]-2,5-diphenyltetrazolium bromide (MTT) was added to each well [8]. The water-soluble MTT was taken up by live cells and reduced by metabolically active cells, partially by the action of dehydrogenase enzymes in mitochondria. The intracellular insoluble purple formazan was quantified, and the absorbance was measured at 590 nm by using an ELISA Reader (Molecular Devices, San Francisco, CA, USA).

### 2.7. Statistics

All data are expressed as means ± standard errors of the means (SEMs) and were analyzed through a one-way analysis of variance. Differences among groups were analyzed through Duncan’s multiple range test (SPSS version 14.0), and *p* < 0.05 was considered significant.

## 3. Results

### 3.1. Determining Potential Biological Functions of Curcumin using the C-Map and CLUE Databases

**Figure 1:**
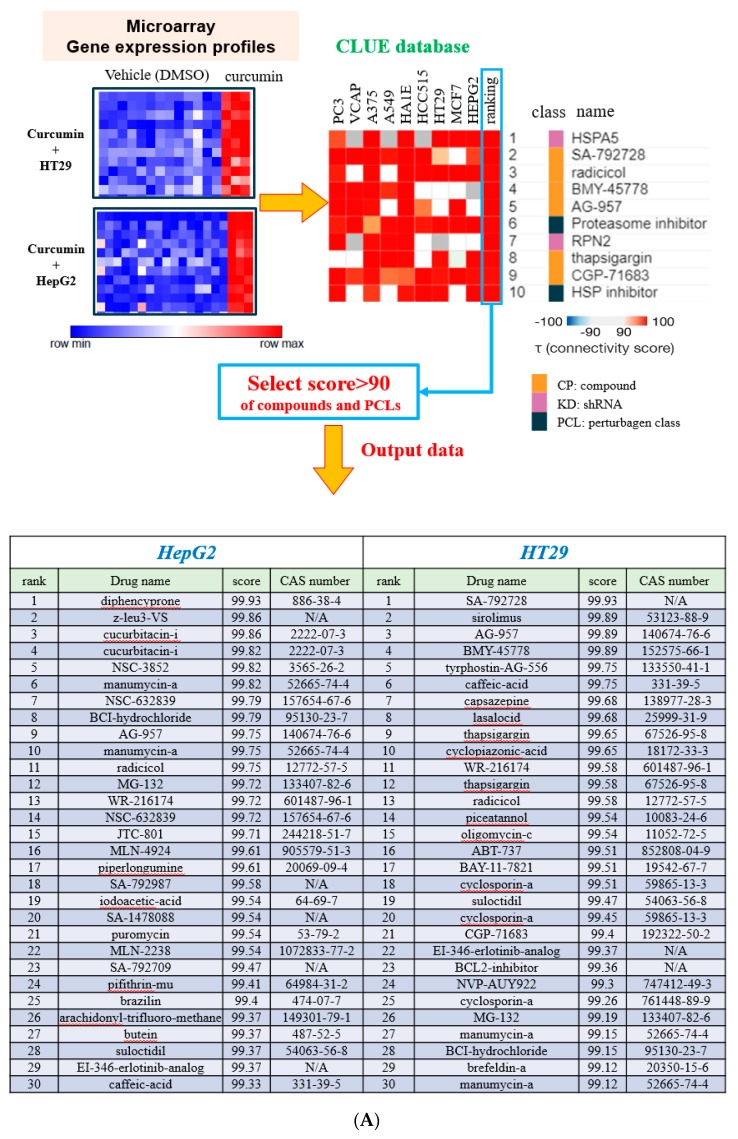

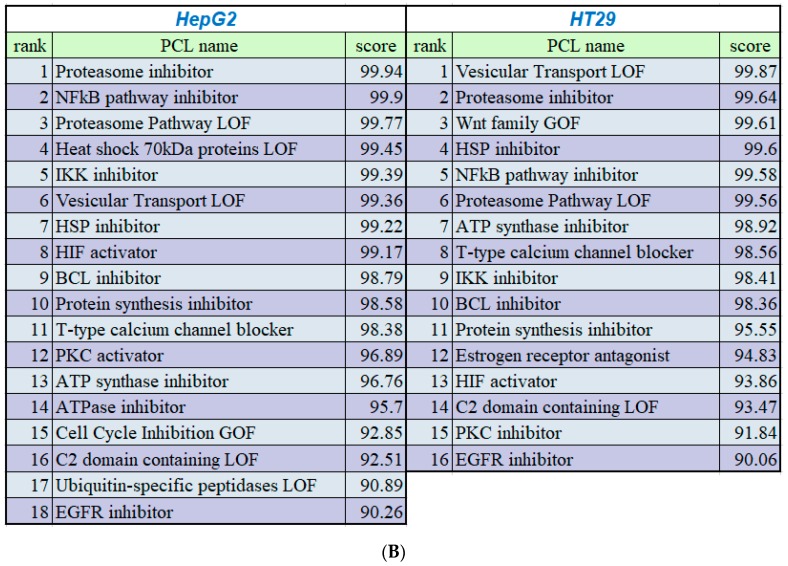
Gene analysis of curcumin. (**A**) The microarray data represent the L1000 gene-expression profiles of HT29 and HepG2 cells treated with curcumin (top, left). Using these gene expression signatures to query the CLUE (C-Map and LINCS (library of integrated network-based cellular signatures) unified environment) database, we obtained compounds or short hairpin RNAs (shRNAs) with similar gene expression signatures stored in CLUE according to the connectivity score (shown in blue). The top 30 compounds are presented at the bottom of the figure. The complete list is provided in supplementary information. Connectivity score was based on the Kolmogorov–Smirnov enrichment statistical evaluation of each gene expression profile. PCLs denotes perturbagen classes identifying groups of compounds by the mechanism of action and identifying groups of genes belonging to the same functional family. (**B**) L1000 array analysis of curcumin using CLUE–PCL.

The microarray analysis of HT29 and HepG2 treated with curcumin yielded two sets of L1000 gene expression profiles that were subsequently incorporated into CLUE [4] and the C-Map [3] to calculate the connectivity score of drugs and PCLs for prediction of similar mechanisms (Figure 1A). Compounds exhibiting similar functions to those of curcumin in HepG2 and HT29 cell lines were revealed by querying the C-Map and CLUE databases. The output data are presented in Figures S3A,B, and more than 90 PCL scores are listed in Figure S3C. The heat maps for both cell lines are listed in Figure S4. To optimize the analysis, the 30 compounds with the highest PCL scores were selected from the CLUE database (Figure 1A), and nine compounds with functions in common with curcumin such as manumycin-a and caffeic acid were obtained (Figure S1A). The molecular targets of these nine compounds are listed in Figure S1E. The PCLs with the scores more than 90 were identified (Figure 1B), and the intersected PCLs revealed that curcumin may have inhibited IκB kinase (IKK) and NF-κB (Figure S1B), again indicating that inhibition of the NF-κB pathway might be a function of curcumin.

The 30 compounds with the highest scores in the C-Map were also selected to represent compounds that may hold gene expression signatures identical to that of curcumin. After the 30 highest-scoring compounds in HepG2 and HT29 cell lines (Figure S1C) had been intersected and these compounds had been further identified according to their mechanisms or biological functions, 18 compounds with functions in common with curcumin, including withaferin A, securinine, and parthenolide, were obtained from the C-Map database (Figure S1D). The results indicated that curcumin may have acted as an inhibitor of IKK and NF-κB and as an activator of TP53.

### 3.2. Validation of Predicted Results Using the Proposed Gene Expression Screening Platform

**Figure 2:**
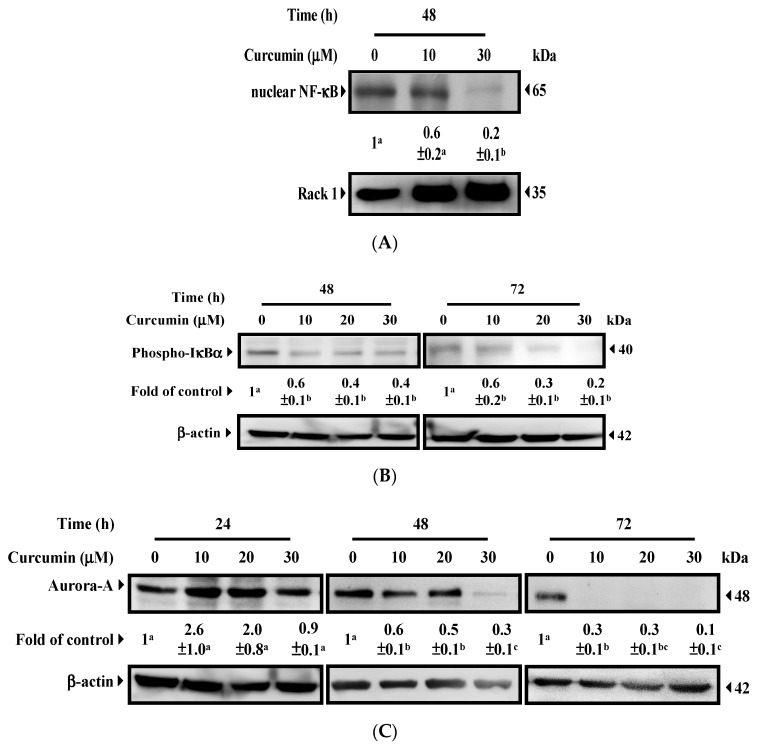

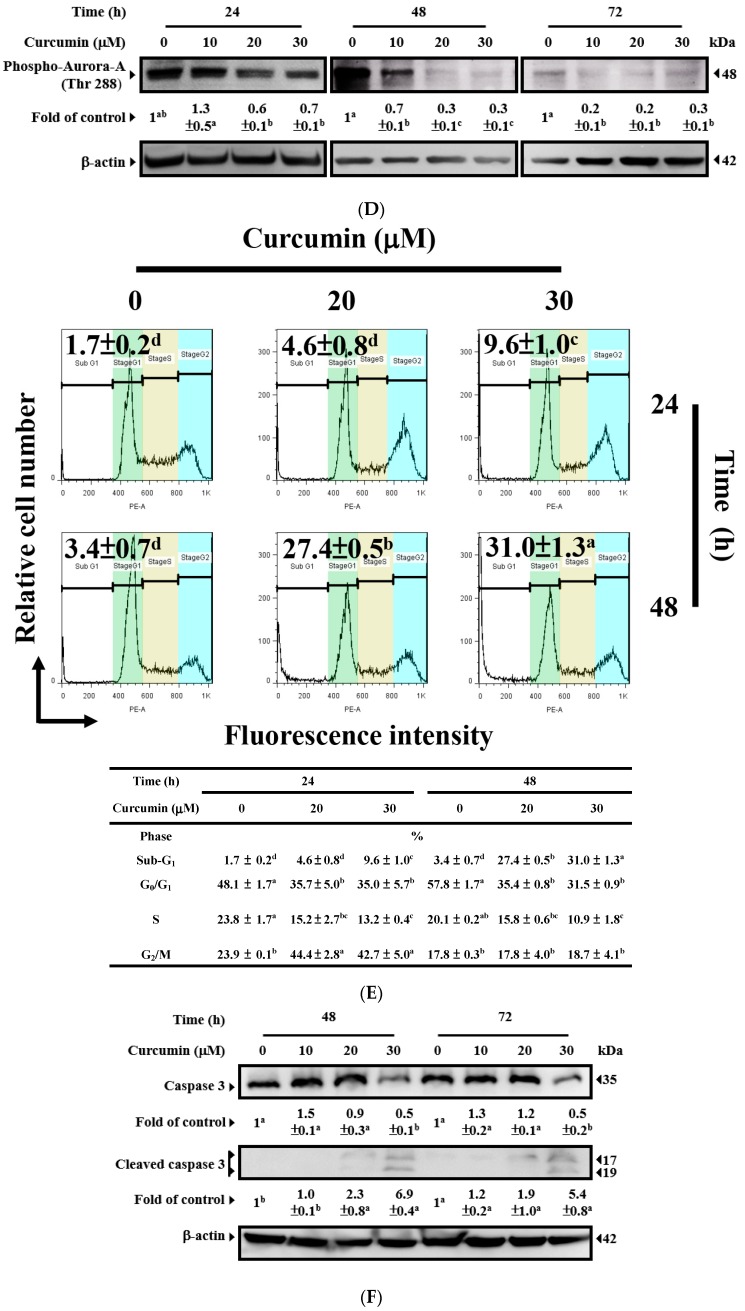

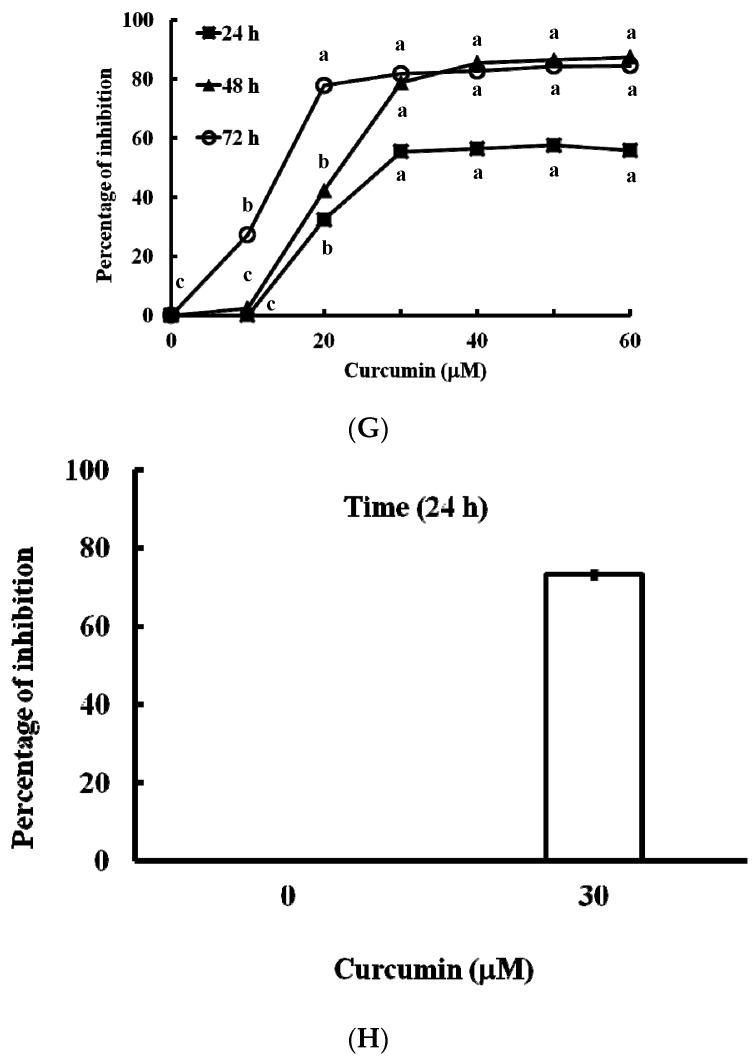
Confirmation of the effects of curcumin on HT29 cells. (**A**) Curcumin reduced nuclear NF-κB expression. Nuclear proteins were prepared for Western blot analysis by using anti-p65 NF-κB antibody. Rack 1 served as a loading control. (**B**) Curcumin suppressed IκB-α phosphorylation. (**C**) Curcumin reduced Aurora-A protein expression. (**D**) Curcumin inhibited Aurora-A kinase activity. After treatment, total cell lysates underwent Western blot analysis with anti-phospho-IκBα, anti-Aurora-A, and anti-phospho-Aurora-A antibodies. β-actin served as a loading control. (**E**) Induction of apoptosis and G2/M arrest. After treatment, HT29 cells were stained with propidium iodide before flow cytometry. The percentages indicate the proportion of apoptotic cells. The percentages of cells at different phases of the cell cycle were also calculated. All data are presented as means ± SEMs. The means in cell cycles without common letters differed; *p* < 0.05. (**F**) Curcumin activated caspase 3. After treatment, total cell lysates underwent Western blot analysis using anti-caspase 3 antibody; β-actin served as a loading control. For Western blot analysis, the intensity of each protein expression band was quantified through densitometry normalization to that of Rack 1 or β-actin, with the control level arbitrarily set to 1. All data are presented as means ± SEMs. Means without common letters differed; *p* < 0.05. (**G**) Growth inhibition of HT29 cells determined using MTT assay. (**H**) Growth inhibition of HT29 cells determined using trypan blue. All data are presented as means ± SEMs. The means at time points without common letters differed; *p* < 0.05.

We predicted that curcumin would inhibit NF-κB (Figure 1). To validate the results predicted using our novel gene expression screening platform, the nuclear expression of NF-κB and its upstream regulators was determined. As shown in Figure 2A, curcumin considerably decreased the nuclear expression of NF-κB. A decrease in the expression of phospho-IκBα (Figure 2B) confirmed that curcumin acted as an inhibitor of IKK and NF-κB. We also predicted that curcumin would activate TP53 (securinine [11]; Figure S1D). Aurora-A phosphorylates p53 and inhibits the DNA binding activity and transactivation activity of p53 [12]. As shown in Figure 2C, the expression of total Aurora-A protein was lower when the HT29 cells were treated with curcumin for 48 and 72 h. A decrease in phospho-Aurora-A expression (Figure 2D) suggested that the activity of Aurora-A kinase had been suppressed [13,14]. These results indicated that curcumin-induced activation of p53 may proceed through the inhibition of Aurora-A expression and kinase activity.

Curcumin was expected to inhibit Abl kinase and mitogen-activated protein (MAP) kinase (Figure S1A). Thiotanib is a tyrosine kinase inhibitor that targets Bcr-Abl. Thiotanib-induced apoptosis was reported in a recent study [15]. Moreover, inhibition of MAP kinase was linked to caspase-3-dependent apoptosis and G2/M arrest [16], and suppression of NF-κB was associated with induction of apoptosis [17]. Curcumin-reduced Aurora-A protein expression (Figure 2C) implies the induction of G/2M arrest [18,19]. As shown in Figure 2E, curcumin triggered apoptosis in a time- and dosage-related manner. The induction of apoptosis was confirmed by the expression of active/cleaved caspase 3 (Figure 2F) [20]. Notably, significant G2/M arrest was observed after 24 h of treatment (Figure 2E). The results strengthened the findings presented in Figure 1, indicating that gene expression profiles provide an opportunity to identify the effects of curcumin. The cytotoxic effect of curcumin was analyzed. As shown in Figure 2G, curcumin suppressed the population growth of HT29 cells in a time- and dosage-related manner. To confirm that the cytotoxic results obtained using MTT assay (which measures functional mitochondrial dehydrogenases as an indicator of overall cellular metabolic activity) had not been influenced by the effect of curcumin on mitochondria [21], the number of viable cells after curcumin treatment was counted after the cells had been stained with trypan blue (Figure 2H). The results indicated that curcumin reduced the population growth of the cells, possibly through the induction of apoptosis and G2/M arrest, both of which could be anticipated by measuring the disturbance of molecules through gene expression profiles.

### 3.3. Identification of Novel Molecules and Pathways Involved in Curcumin-Suppressed NF-κB

**Figure 3:**
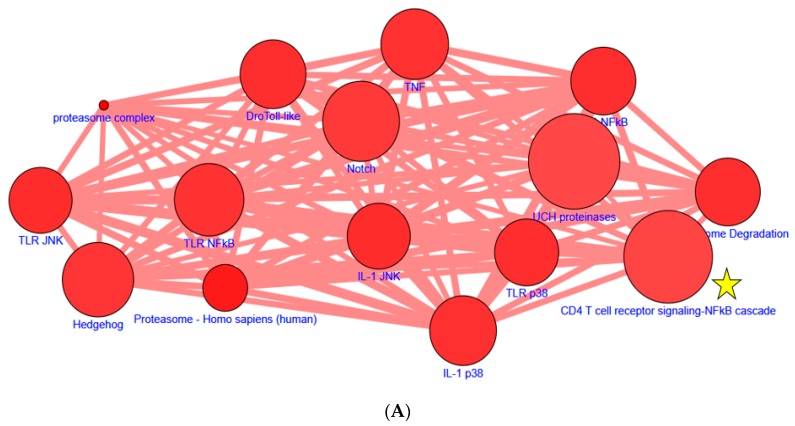

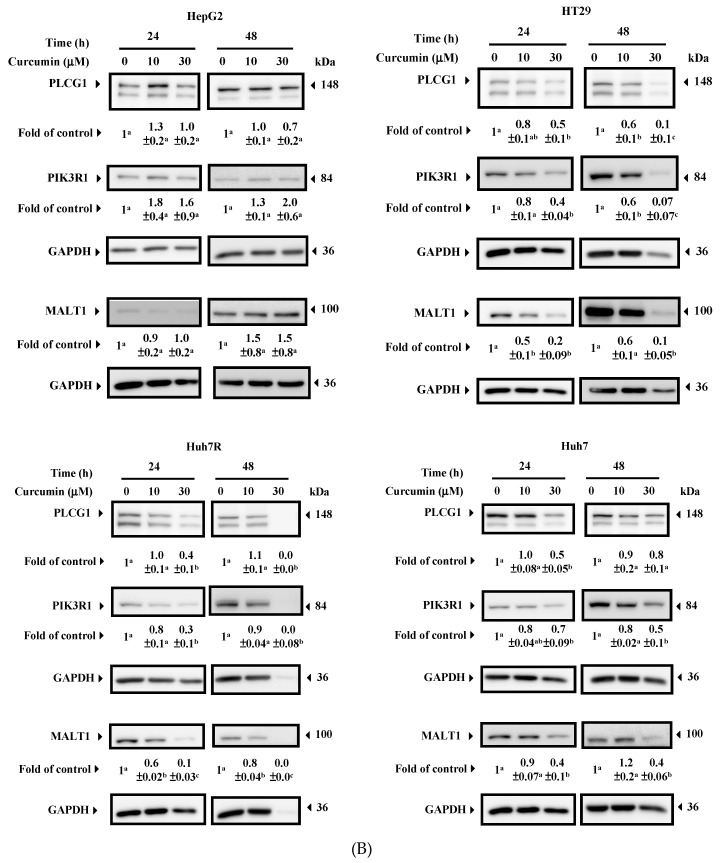

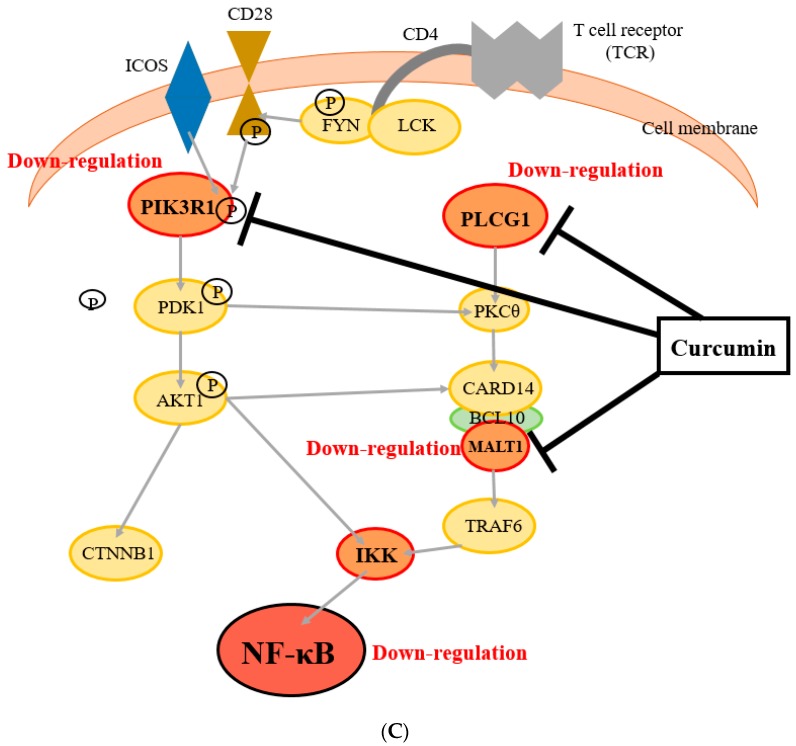
Using ConsensusPathDB (CPDB) to identify novel modulation by curcumin. (**A**) Prediction of high correlation pathways. Genes common between two sample sets were used to query CPDB in order to predict the pathways in which these genes were likely participating. The network shows the 20 highest-scoring pathways from CPDB. The size of each dot denotes the entity number of genes in the pathway. The line between two dots was calculated by the function of these two pathways to indicate the number of genes overlapping said pathways. The breadth of the line denotes the strength of the correlation between two dots. The yellow star denotes the pathway selected for validation. Details regarding the analysis of these 43 pathways are listed in Figure S2. (**B**) Curcumin reduced MALT1, PIK3R1, and PLCG1 expression. Most of the HT29 and Huh7R cells were dead after 48 h. The intensity of each protein expression band was quantified through densitometry normalization to that of GAPDH, with the control level arbitrarily set to 1. All data are presented as means ± SEMs. Means without common letters differed; *p* < 0.05. (**C**) Proposed mechanism of curcumin on the CD4-T-cell-receptor-signaling NF-κB pathway.

To further explore our observation that curcumin functions as an inhibitor (Figure 1 and Figure S1), the molecules involved in NF-κB inhibition were determined. We further analyzed the two sets of common short hairpin RNAs (shRNAs) in CPDB and predicted the specific pathways involved (Figure 3A). The 43 pathways based on *p* values are listed in Figure S2 (*p* value < 0.001). Tumor necrosis factor (TNF) has been frequently reported to be involved in a key pathway regulating NF-κB. The CD4-T-cell-receptor-signaling NF-κB cascade was selected because this pathway is not often studied. Western blotting analysis was performed to examine this cascade. Other pathways with lower *p* values were also examined; the expression of the molecules involved was not significantly altered (data not shown). The expression of PLCG1, PIK3R1, and MALT1 was evaluated after 24 and 48 h of curcumin treatment. The results indicated that the expression of PLCG1, PIK3R1, and MALT1 was suppressed when HT29, Huh7R, and Huh7 cells received curcumin (30 μM) for 24 and 48 h (Figure 3B). These results indicated that the CLUE and CPDB databases had enabled us to accurately predict that curcumin might act as an NF-κB inhibitor by suppressing the CD4-T-cell-receptor-signaling NF-κB cascade pathway (Figure 3C).

## 4. Discussion

Although curcumin has been the subject of more than 100 clinical trials with results demonstrating that it is both safe and effective for protecting against multiple chronic diseases and cancers [2,22], curcumin can interact with numerous proteins, and the novel signaling targets of this pleiotropic phytonutrient may yield both pharmaceutical benefits and unwanted side effects such as the induction of gall bladder contraction (20–40 mg), reduction of chemosensitivity [2], and having abdominal pain (200 mg) [23]. The most common side effects of curcumin are nausea and diarrhea [24]. Increases in serum alkaline phosphatase and lactate dehydrogenase have also been reported [25]. Foods thought to be beneficial to health are sometimes consumed in amounts that may actually produce negative health outcomes. Exposure to supernutritional levels of phytochemicals or nutraceuticals may induce various physiological phenomena, and thus the unknown molecular actions of these compounds require urgent identification; a gene expression screening platform offers this capability.

The C-Map (invented by the Broad Institute of the Massachusetts Institute of Technology and Harvard University) contains gene expression profiles with four specific human cancer cell lines (breast cancer MCF7, prostate cancer PC3, leukemia HL60, and melanoma SKMEL5) treated with small bioactive molecules, including approximately 900 United States Food and Drug Administration (FDA)-approved drugs [3,26]. Lamb et al. [3] postulated that disease-associated gene signatures can be compared with C-Map compound signature profiles to reveal potential compounds, even when profiles from other cell lines are used. Notably, several other cancers, including acute leukemia, colon cancer, hepatocellular carcinoma, neuroblastoma, non-small-cell lung cancer, and renal cell carcinoma, can be employed in the C-Map system to search for possible molecular mechanisms.

Although the bioavailability of oral curcumin is low, several studies have observed that curcumin exhibits antitumor activities against various cancers. The lower incidences of cancer and cancer death, including colon cancer [27] and other cancers [28], in India compared with the USA might be at least partially attributable to the relatively high dietary intake of spices such as turmeric (i.e., curcumin) in South Asia. Curcumin activity in humans may be caused by accumulation of the compound and its metabolites in cells as a result of daily exposure. Moreover, curcumin is more widely distributed throughout the gastrointestinal tract compared with that situated remotely from the site of absorption [29]. The anticarcinogenic effect of curcumin has been reported [30,31] in vitro and *in vivo*, and phase III human clinical trials for colon cancer are ongoing [32].

Our novel screening platform effectively identified the molecular mechanisms of a phytochemical [33]. The gene expression profiles of cancer cells treated with or without a given phytochemical indicate which genes are up- and down-regulated by said phytochemical. The C-Map can be queried to identify specific genes that are up- and down-regulated by small molecules, including FDA-approved drugs. If a compound exhibits a similar gene profile to that of a FDA-approved drug in the C-Map, then the compound may have similar molecular mechanisms to said drug. CLUE is similar to the C-Map but considerably larger, with more than 1.1 million L1000 profiles. In the present study, similarity scores for compounds in CLUE were obtained to identify their molecular actions.

Curcumin is known to exert strong anti-inflammatory effects by interrupting NF-κB signaling at multiple levels. Most related studies have investigated the effect of curcumin on TNF signaling through the IKK complex [34,35]. As expected, regulation of TNFα by curcumin was predicted through CPDB analysis. Notably, the use of gene expression profiles to query databases such as CLUE and CPDB in the present study enabled us to identify novel molecules and pathways regulated by curcumin; down-regulation of PLCG1, PIK3R1, and MALT1 was involved in a novel CD4-T-cell-receptor-signaling NF-κB cascade. Dűwel et al. reported that A20 regulated the strength and duration of the IKK/NF-κB response upon TCR/CD28 costimulation [36]. A20 was demonstrated as an ubiquitin-editing enzyme by inactive MALT1, leading to sustained interaction between MALT1 and the IKK complex. A20 inhibited the CD4-T-cell-receptor-signaling NF-κB cascade. No studies have investigated the CD4-T-cell-receptor-signaling NF-κB cascade regulated by curcumin. The CD4-T-cell-receptor-signaling NF-κB was selected in the present study because this pathway is rarely researched. Therefore, using big data analysis enabled us to uncover novel signaling pathways and in turn research molecular targets for disease therapy and prevention. However, without a T-cell receptor being activated through binding to a MHC-antigen presenting cell (or by using some compounds able to induce partial or full TCR activation), investigating the TCR-mediated changes in signaling and the supposed effects on downstream-signaling molecules is impossible. The present study partially overlapped with signaling described for immune cells, but the most marked benefit of this research is the implication of the findings in the field of cancer research, namely that of colon cancer. Therefore, the use of T-lymphocytes with the CD4 phenotype to fully uncover a CD4+ T-cell receptor mediated signaling pathway is necessary.

By using gene expression profiling to query LINCS, we identified the phytochemical justicidin A as an autophagy inducer [33]. Discovering and developing new drugs is not only time consuming but also costly. Decades may be required to introduce a new drug on the market, and many seemingly promising drugs fail clinical trials. Since its introduction in 2006, the C-Map has served as a powerful tool with numerous applications, including the repurposing of drugs. Thus, the C-Map facilitates both drug discovery and drug repurposing. By querying the C-Map through profiling, we repurposed existing FDA-approved antipsychotic drugs (trifluoperazine, thioridazine) and one antibiotic (antimycin A) to overcome drug resistance in lung cancer [37,38] and glioblastoma [39] stem-like cells both in vitro and *in vivo*. Furthermore, the present novel gene expression screening platform was previously used to analyze the complex therapeutic effects of traditional Chinese medicine formulae such as that for PG2 [40].

Although the proposed method of drug discovery enables researchers to determine numerous potential biological functions and mechanism of actions (MOAs) of unknown compounds, it is not without drawbacks. For example, in the CLUE database, a maximum of 150 up- and down-regulated genes can be uploaded, and thus only the top 150 up- and down-regulated genes can be selected to query the database. However, although these 150 genes may represent only a small proportion of the whole population in some cases, the results are reliable because these genes are ranked highest in terms of prevalence. Despite this shortcoming, analyses of PCLs predominantly focus on compounds, and thus this method is still considered reliable. In addition, for a small molecule such as curcumin, interrupting many crucial molecular signaling pathways might be unsuitable for application in human disease therapy. Many targets lead to side effects and adverse outcomes in patients, and thus their use should be cautious in an era of considerably superior medicines (i.e., biologics and antibodies).

## 5. Conclusions

We used the known molecular actions of curcumin (inhibition of NF-κB and Aurora-A and induction of G2/M arrest and apoptosis) as positive controls to validate our novel gene expression screening platform and applied said platform to identify novel pathways (inhibition of PLCG1, PIK3R1, and MALT1 in the CD4-T-cell-receptor-signaling NF-κB cascade pathway) in curcumin-suppressed NF-κB. Therefore, the exploratory modality provided by the C-Map enables researchers to discern connections among diseases, genes, and chemical expression profiles and facilitates the identification of novel molecular actions (including positive benefits and negative side effects) of phytochemicals and nutraceuticals.

## Supplementary Materials

The following are available online at https://www.mdpi.com/2072-6643/11/6/1397/s1, Figure S1. Intersection compounds and analysis of curcumin by using the CLUE and C-Map. Figure S2. Prediction of highly correlated pathways. Figure S3. The output data of compounds (CP) and PCL were analyzed from CLUE (score ≥ 90). Figure S4. The heat maps for HepG2 and HT29.
