# Supplementary material for: Using the Pleiotropic Characteristics of Curcumin to Validate the Potential Application of a Novel Gene Expression Screening Platform
Source: Nutrients. 2019 Jun 21;11(6):1397. doi: 10.3390/nu11061397 (PMC6627093; doi:10.3390/nu11061397)
Supplement: Supplementary file 1 [file nutrients-11-01397-s001.pdf]

Supplementary Materials

Identification of Compounds

The connectivity map concept (C-Map) is based on gene expression profiles, also known as gene fingerprints, and is used to analyze similar effects of compounds and to find drugs for treating diseases [1]. The gene expression profiles in both the C-Map and the CLUE [2] websites were derived from the treatment of human cells with thousands of drugs. Therefore, the gene expression signatures of interest in any induced or organic cell state could be compared with one another to determine similar mechanisms or reverse signatures of drugs and shRNA. Pattern-matching algorithms were used to score each gene expression profile and provide strength of enrichment through query signatures. The results were ranked by "connectivity score (τ)"; a positive score of a signature denoted a similar effect, whereas a negative score indicated a contrary effect. A τ of 90 indicated that only 10% of all perturbations exhibited strong connectivity to the query [2].

Methodology of perturbagen classes (PCLs)

To render the CLUE database relatively easy for users to quickly find the mechanism of action (MOA) of a target drug, codifying the class-level annotation required considerable effort. MOAs were adopted to identify groups of compounds with distinct chemical structures, and genetic perturbagens were grouped on the basis of their belonging to the same

gene family or being commonly targeted by the same compounds. Ultimately, CLUE named PCLs for their class-level annotations and further connected these cognate class members according to the results of L1000 connectivity analyses to predict the mechanism [2].

A

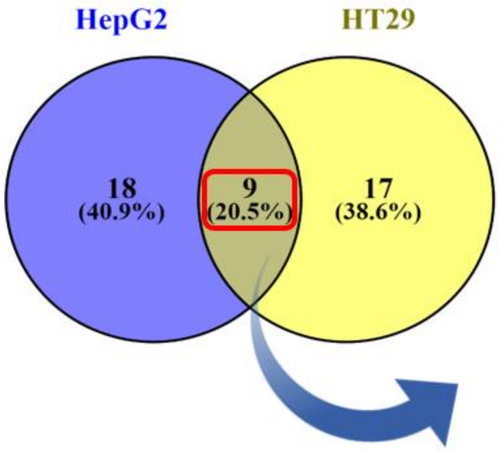

Compound name	CLUE description	CAS number
manumycin-a	farnesyltransferase inhibitor, NFkB pathway inhibitor, ras farnesyltransferase inhibitor	52665-74-4
BCI-hydrochloride	MAP kinase phosphatase inhibitor, protein phosphatase inhibitor	95130-23-7
AG-957	Abl kinase inhibitor, protein tyrosine kinase inhibitor	140674-76-6
radicicol	HSP inhibitor, ATP citrase lyase inhibitor, MAP kinase inhibitor, opioid receptor ligand, pyruvate dehydrogenase kinase inhibitor	12772-57-5
MG-132	proteasome inhibitor	133407-82-6
WR-216174	CDK inhibitor, PFMRK inhibitor	601487-96-1
suloctidil	adrenergic receptor antagonist, platelet aggregation inhibitor, vasodilator	54063-56-8
EI-346-erlotinib-analog	epidermal growth factor receptor (EGFR) inhibitor	N/A
caffeic-acid	lipoxygenase inhibitor, HIV integrase inhibitor, NFkB pathway inhibitor, nitric oxide production inhibitor, PPAR receptor modulator, tumor necrosis factor production inhibitor	331-39-5

B

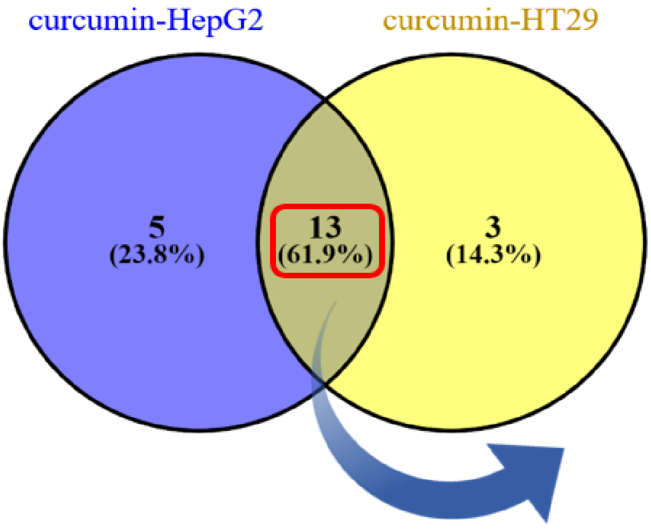

Proteasome inhibitor
NFkB pathway inhibitor
Proteasome Pathway LOF
IKK inhibitor
Vesicular Transport LOF
HSP inhibitor
HIF activator
BCL inhibitor
Protein synthesis inhibitor
T-type calcium channel blocker
ATP synthase inhibitor
C2 domain containing LOF
EGFR inhibitor

C

HepG2				*HT29*			
rank	cmap name	enrichment	P-value	rank	cmap name	enrichment	P-value
1	MG-262	0.998	0	1	phenoxybenzamine	0.996	0
2	phenoxybenzamine	0.995	0	2	5224221	0.982	0.00062
3	5224221	0.993	0.00004	3	5182598	0.978	0.00082
4	withaferin A	0.987	0	4	lomustine	0.977	0
5	terfenadine	0.983	0.00004	5	parthenolide	0.976	0
6	calmidazolium	0.983	0.00054	6	MG-262	0.974	0.00004
7	piperlongumine	0.982	0.00062	7	thapsigargin	0.973	0.00004
8	1,4-chrysenequinone	0.977	0.00087	8	piperlongumine	0.972	0.00133
9	puromycin	0.971	0	9	withaferin A	0.968	0
10	menadione	0.97	0.00165	10	calmidazolium	0.965	0.00211
11	astemizole	0.968	0	11	ionomycin	0.958	0.0001
12	lomustine	0.968	0	12	semustine	0.937	0.00002
13	disulfiram	0.959	0	13	thiostrepton	0.937	0.00002
14	anisomycin	0.954	0	14	1,4-chrysenequinone	0.937	0.00765
15	5182598	0.953	0.00392	15	STOCK1N-35215	0.93	0.00062
16	pyrvinium	0.944	0	16	clotrimazole	0.929	0
17	suloctidil	0.94	0	17	butein	0.928	0.00984
18	securinine	0.934	0.00002	18	STOCK1N-35696	0.922	0.01195
19	parthenolide	0.93	0.00004	19	securinine	0.916	0.00004
20	butein	0.93	0.00942	20	astemizole	0.909	0.00004
21	rottlerin	0.929	0.00066	21	azacitidine	0.903	0.00182
22	mefloquine	0.926	0.00002	22	norcyclobenzaprine	0.902	0.0001
23	5155877	0.926	0.00004	23	suloctidil	0.893	0.00012
24	ouabain	0.921	0.00004	24	terfenadine	0.89	0.00252
25	mebendazole	0.917	0.00004	25	16,16-dimethylprostaglandin E2	0.889	0.00262
26	thiostrepton	0.915	0.00004	26	nifuroxazide	0.876	0.00034
27	protriptyline	0.913	0.00004	27	noretynodrel	0.873	0.00036
28	digoxin	0.913	0.00006	28	irinotecan	0.868	0.00417
29	STOCK1N-35215	0.91	0.00154	29	5230742	0.867	0.0364
30	proscillaridin	0.909	0.00156	30	puromycin	0.861	0.0005

D

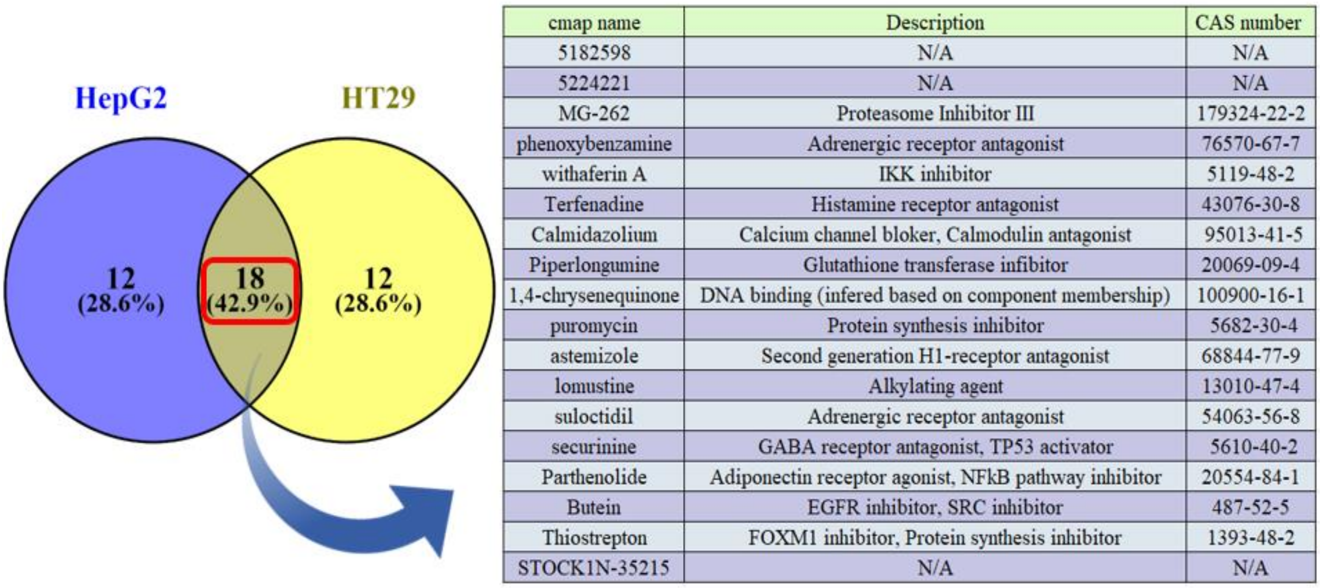

cmap name	Description	CAS number
5182598	N/A	N/A
5224221	N/A	N/A
MG-262	Proteasome Inhibitor III	179324-22-2
phenoxybenzamine	Adrenergic receptor antagonist	76570-67-7
withaferin A	IKK inhibitor	5119-48-2
Terfenadine	Histamine receptor antagonist	43076-30-8
Calmidazolium	Calcium channel bloker, Calmodulin antagonist	95013-41-5
Piperlongumine	Glutathione transferase infibitor	20069-09-4
1,4-chrysenequinone	DNA binding (infered based on component membership)	100900-16-1
puromycin	Protein synthesis inhibitor	5682-30-4
astemizole	Second generation H1-receptor antagonist	68844-77-9
lomustine	Alkylating agent	13010-47-4
suloctidil	Adrenergic receptor antagonist	54063-56-8
securinine	GABA receptor antagonist, TP53 activator	5610-40-2
Parthenolide	Adiponectin receptor agonist, NFkB pathway inhibitor	20554-84-1
Butein	EGFR inhibitor, SRC inhibitor	487-52-5
Thiostrepton	FOXM1 inhibitor, Protein synthesis inhibitor	1393-48-2
STOCK1N-35215	N/A	N/A

E

HepG2			
rank	drug name	target	score
1	diphencyprone	IL-17 , IL-4	99.93
2	z-leu3-VS	PGPH	99.86
3	cucurbitacin-i	JAK2, STAT3	99.86
4	NSC-3852	HDAC1	99.82
5	cucurbitacin-i	JAK2, STAT3	99.82
6	manumycin-a	FNTA, IKBKB	99.82
7	NSC-632839	USP2, USP7, SENP2, USP1	99.79
8	BCI-hydrochloride	DUSP1, DUSP6	99.79
9	radicicol	ACLY, DLAT, HSP90AB1, HSP90B1, MAP3K7, OPRM1, PDK3	99.75
10	manumycin-a	FNTA, IKBKB	99.75
11	AG-957	ABL1, EGFR	99.75
12	MG-132	PSMB1	99.72
13	NSC-632839	USP2, USP7, SENP2, USP1	99.72
14	WR-216174	Pfmrk	99.72
15	JTC-801	OPRL1	99.71
16	piperlongumine	NF-κB, PI3K/AKT	99.61
17	MLN-4924	NAE1, UBA3	99.61
18	SA-792987	WEE1	99.58
19	MLN-2238	PSMB1	99.54
20	SA-1478088	MMP12, MMP14, MMP2, MMP8, MMP9	99.54
21	puromycin	NHP2L1, RPL10L, RPL11, RPL13A, RPL15, RPL19, RPL23, RPL23A, RPL26L1, RPL3, RPL37, RPL8, RSL24D1	99.54
22	iodoacetic-acid	GAPDH	99.54
23	SA-792709	RARA, RARB	99.47
24	pifithrin-mu	HSPA1A, TP53	99.41
25	brazilin	NF-κB	99.4
26	EI-346-erlotinib-analog	EGFR	99.37
27	arachidonyl-trifluoro-methane	PLA2G4A	99.37
28	suloctidil	SMPD1	99.37
29	butein	ACE, CXCL8, IL6, SIRT1, SRD5A1, SRD5A2, TNF	99.37
30	caffeic-acid	ALOX5, MIF, RELA, TNF	99.33

HT29			
rank	drug name	target	score
1	SA-792728	SPHK1, VCP	99.93
2	BMY-45778	PTGIR	99.89
3	AG-957	ABL1, EGFR	99.89
4	sirolimus	MTOR, FKBP1A, CCR5, FGF2	99.89
5	caffeic-acid	ALOX5, MIF, RELA, TNF	99.75
6	tyrphostin-AG-556	EGFR	99.75
7	lasalocid	LC-3II, PARP	99.68
8	capsazepine	TRPV1, TRPV4	99.68
9	cyclopiazonic-acid	ATP2A1	99.65
10	thapsigargin	ATP2A1	99.65
11	radicicol	ACLY, DLAT, HSP90AB1, HSP90B1, MAP3K7, OPRM1, PDK3	99.58
12	thapsigargin	ATP2A1	99.58
13	WR-216174	Pfmrk	99.58
14	oligomycin-c	ATP5A1	99.54
15	piceatannol	ATP5A1, ATP5B, ATP5C1, IRF3, PTGS2, SIRT1, SYK, TYR	99.54
16	cyclosporin-a	PPIA, ABCB11, CAMLG, CYP3A5, CYP3A7, FPR1, PPID, PPIF, PPP3CA, PPP3R2, SLC10A1, SLCO1B1, SLCO1B3	99.51
17	ABT-737	BCL2, BCL2L1, BCL2L2	99.51
18	BAY-11-7821	RELA	99.51
19	suloctidil	SMPD1	99.47
20	cyclosporin-a	PPIA, ABCB11, CAMLG, CYP3A5, CYP3A7, FPR1, PPID, PPIF, PPP3CA, PPP3R2, SLC10A1, SLCO1B1, SLCO1B3	99.45
21	CGP-71683	NPY5R	99.4
22	EI-346-erlotinib-analog	EGFR	99.37
23	BCL2-inhibitor	BCL2	99.36
24	NVP-AUY922	HSP90AA1, HSP90AA2, HSP90AB1	99.3
25	cyclosporin-a	PPIA, ABCB11, CAMLG, CYP3A5, CYP3A7, FPR1, PPID, PPIF, PPP3CA, PPP3R2, SLC10A1, SLCO1B1, SLCO1B3	99.26
26	MG-132	PSMB1	99.19
27	BCI-hydrochloride	DUSP1, DUSP6	99.15
28	manumycin-a	FNTA, IKBKB	99.15
29	brefeldin-a	ARF1, ARFGEF1, ARFGEF2, CYTH2, GBF1, SAR1A	99.12
30	manumycin-a	FNTA, IKBKB	99.12

Figure S1. Intersection compounds and analysis of curcumin using the CLUE and C-Map. The L1000 gene expression data of HT29 and HepG2 cells treated with curcumin were analyzed by CLUE (https://clue.io/) (**A** and **B**) and C-Map (https://portals.broadinstitute.org/cmap/) (**C** and **D**), respectively. (**A** and **B**) Compounds and PCLs from HT29 and HepG2 were first predicted by CLUE and then subjected to intersection. The 9 intersected compounds (**A**) and 13 PCLs (**B**) are shown. (**C**) The same L1000 gene expression data of curcumin-treated HT29 and HepG2 were analyzed using the C-Map. (**D**) Intersected compounds are shown and annotated. Because C-Map does not provide the drug information, we have annotated these drugs via several public databases. To the best of our knowledge, there are no available annotations for some drugs, which are labelled N/A, accordingly. (**E**) The list, which was provided by the CLUE database, shows the targets of the 30 highest-scoring compounds predicted by CLUE. Compounds without CLUE annotations are labelled N/A.

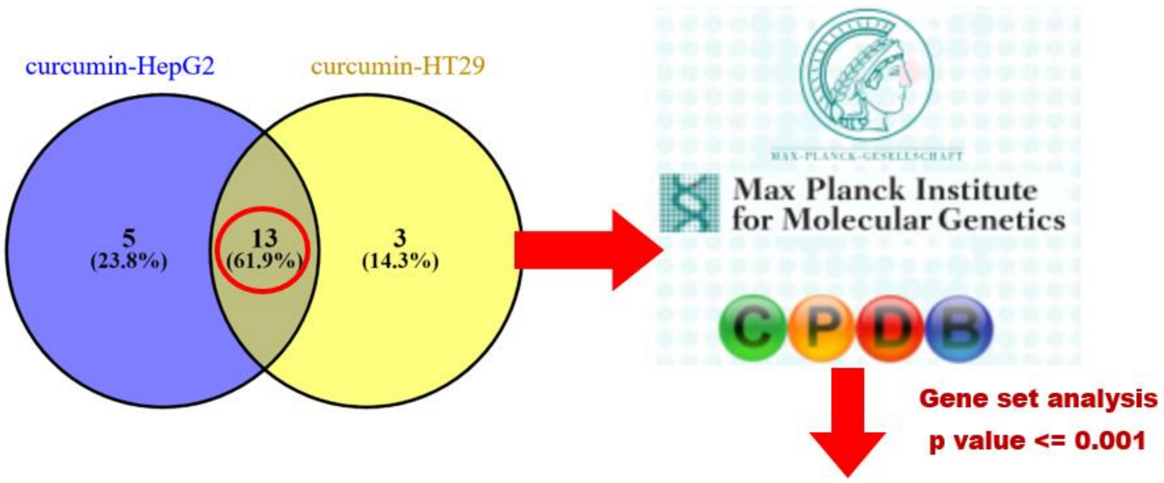

rank	pathway name	candidates/total	p-value	q-value	pathway source
1	proteasome complex	5 (20.8%)	0.0000000	0.0000000	BioCarta
2	Proteasome - Homo sapiens (human)	5 (11.1%)	0.0000000	0.0000000	KEGG
3	TLR JNK	5 (8.1%)	0.0000000	0.0000000	INOH
4	IL-1 JNK	5 (8.1%)	0.0000000	0.0000000	INOH
5	TLR p38	5 (7.9%)	0.0000000	0.0000000	INOH
6	IL-1 NFkB	5 (7.8%)	0.0000000	0.0000000	INOH
7	Proteasome Degradation	5 (7.8%)	0.0000000	0.0000000	Wikipathways
8	DroToll-like	5 (7.7%)	0.0000000	0.0000000	INOH
9	IL-1 p38	5 (7.6%)	0.0000000	0.0000000	INOH
10	TNF	5 (7.5%)	0.0000000	0.0000000	INOH
11	TLR NFkB	5 (7.2%)	0.0000000	0.0000000	INOH
12	Hedgehog	5 (6.9%)	0.0000000	0.0000000	INOH
13	Notch	5 (6.4%)	0.0000000	0.0000000	INOH
14	CD4 T cell receptor signaling-NFkB cascade	5 (5.2%)	0.0000000	0.0000000	INOH
15	UCH proteinases	5 (4.9%)	0.0000000	0.0000000	Reactome
16	TGF-beta super family signaling pathway canonical	5 (4.3%)	0.0000000	0.0000000	INOH
17	Wnt Canonical	5 (4.2%)	0.0000000	0.0000000	INOH
18	Wnt Mammals	5 (4.2%)	0.0000000	0.0000000	INOH
19	CD4 T cell receptor signaling	5 (3.8%)	0.0000000	0.0000001	INOH
20	B cell receptor signaling	5 (3.7%)	0.0000000	0.0000001	INOH
21	Ub-specific processing proteases	5 (2.3%)	0.0000003	0.0000008	Reactome
22	Neddylation	5 (2.1%)	0.0000004	0.0000011	Reactome
23	Post-translational protein modification	8 (0.6%)	0.0000006	0.0000014	Reactome
25	JAK STAT pathway and regulation	5 (1.6%)	0.0000017	0.0000037	INOH
26	Metabolism of proteins	8 (0.4%)	0.0000104	0.0000220	Reactome
27	COPI-dependent Golgi-to-ER retrograde traffic	3 (3.7%)	0.0000282	0.0000574	Reactome
28	COPI-mediated anterograde transport	3 (3.6%)	0.0000292	0.0000574	Reactome
29	Glycosphingolipid biosynthesis - neolactoseries	2 (12.5%)	0.0000636	0.0001210	EHMN
30	Golgi-to-ER retrograde transport	3 (2.6%)	0.0000795	0.0001440	Reactome
31	Glycosphingolipid biosynthesis - globoseries	2 (11.1%)	0.0000810	0.0001440	EHMN
32	Glycosphingolipid biosynthesis - ganglioseries	2 (9.5%)	0.0001110	0.0001910	EHMN
33	ER to Golgi Anterograde Transport	3 (2.2%)	0.0001300	0.0002170	Reactome
34	Vitamin B9 (folate) metabolism	2 (7.7%)	0.0001720	0.0002780	EHMN
35	Proteoglycan biosynthesis	2 (6.7%)	0.0002290	0.0003600	EHMN
36	Transport to the Golgi and subsequent modification	3 (1.8%)	0.0002380	0.0003640	Reactome
37	Prostaglandin formation from arachidonate	2 (5.9%)	0.0002950	0.0004390	EHMN
38	Intra-Golgi and retrograde Golgi-to-ER traffic	3 (1.6%)	0.0003220	0.0004660	Reactome
39	O-Glycan biosynthesis	2 (5.4%)	0.0003500	0.0004940	EHMN
40	N-Glycan biosynthesis	2 (4.8%)	0.0004520	0.0006210	EHMN
41	Galactose metabolism	2 (4.5%)	0.0004960	0.0006650	EHMN
42	Aminosugars metabolism	2 (3.9%)	0.0006660	0.0008720	EHMN
43	C21-steroid hormone biosynthesis and metabolism	2 (3.5%)	0.0008320	0.0010600	EHMN

Figure S2. Prediction of highly correlated pathways. Genes that were in two sets were used to query CPDB in order to predict the pathways in which these genes were likely participating. The Venn diagram shows two intersecting PCLs (curcumin-treated HT29 and HepG2). We focused on the intersection results indicated by red circles; the results contained 13 PCLs, including 10 compounds and 3 shRNA (Supplementary Figure S1B). We employed shRNA gene lists, including *PSMB5*, *PSMA1*, *PSMA3*, *PSMB1*, *PSMB2*, *COPA*, *COPB2*, *COPZ1*, *UVRAG*, *C2CD2*, and *RAB11FIP2*, to query CPDB in order to analyze interaction network modules, biochemical pathways, and functional information. A total of 43 prediction pathways, which are indicated at the bottom of the figure, were identified according to analysis using the CPDB database ($p < 0.001$), and we analyzed the CD4-T-cell-receptor-signaling NF-κB cascade for further validation (highlighted in yellow).

A

HepG2						
rank	name	belongs (PCL)	pc	median_score	ts_pc	score
1	diphencyprone		62.50, 62.50	99.5	1.84, 0.66	99.93
2	z-leu3-VS	Proteasome inhibitor	100.00, 100.00	99.88	3.00, 0.82	99.86
3	cucurbitacin-i		100.00, 80.00	99.85	2.92, 0.80	99.86
4	NSC-3852	HDAC inhibitor	100.00, 100.00	99.79	2.16, 0.67	99.82
5	cucurbitacin-i		100.00, 100.00	99.78	2.09, 0.45	99.82
6	manumycin-a		80.00, 80.00	99.75	3.15, 0.77	99.82
7	NSC-632839		100.00, 100.00	99.92	2.25, 0.61	99.79
8	BCI-hydrochloride		70.00, 60.00	99.78	2.47, 0.52	99.79
9	radicicol	HSP inhibitor	100.00, 90.00	99.83	2.09, 0.76	99.75
10	manumycin-a	NFkB pathway inhibitor	80.00, 80.00	99.76	2.32, 0.57	99.75
11	AG-957		80.00, 80.00	99.76	1.59, 0.56	99.75
12	MG-132	Proteasome inhibitor	100.00, 100.00	99.83	2.30, 0.69	99.72
13	NSC-632839		80.00, 80.00	99.81	3.01, 0.78	99.72
14	WR-216174		70.00, 70.00	99.24	1.34, 0.45	99.72
15	JTC-801		100.00, 83.33	99.43	2.92, 0.63	99.71
16	piperlongumine		90.00, 90.00	99.71	2.11, 0.70	99.61
17	MLN-4924		80.00, 80.00	99.35	2.53, 0.74	99.61
18	SA-792987	PKC inhibitor	80.00, 60.00	99.04	1.32, 0.30	99.58
19	MLN-2238	Proteasome inhibitor	100.00, 100.00	99.81	2.44, 0.85	99.54
20	SA-1478088		90.00, 80.00	99.52	2.35, 0.81	99.54
21	puromycin		90.00, 90.00	99.43	2.26, 0.58	99.54
22	iodoacetic-acid		55.56, 55.56	98.2	1.67, 0.34	99.54
23	SA-792709		100.00, 90.00	99.68	1.50, 0.55	99.47
24	pifithrin-mu		60.00, 50.00	97.16	1.05, 0.24	99.41
25	brazilin		80.00, 70.00	99.33	1.50, 0.50	99.4
26	EI-346-erlotinib-analog		60.00, 60.00	99.15	1.74, 0.51	99.37
27	arachidonyl-trifluoro-methane		80.00, 80.00	99.06	1.40, 0.15	99.37
28	suloctidil		88.89, 66.67	97.92	2.07, 0.56	99.37
29	butein		60.00, 50.00	97.89	1.52, 0.56	99.37
30	caffeic-acid		50.00, 50.00	94.1	1.24, 0.43	99.33
31	BNTX		100.00, 100.00	99.69	2.36, 0.59	99.3
32	kinetin-riboside		90.00, 90.00	99.4	2.90, 0.75	99.3
33	auranofin	NFkB pathway inhibitor	40.00, 40.00	62.31	2.74, 0.50	99.28
34	parthenolide	NFkB pathway inhibitor	90.00, 90.00	99.51	2.27, 0.65	99.26
35	thiostrepton	Protein synthesis inhibitor	90.00, 90.00	99.23	1.86, 0.31	99.26
36	SSR-69071		80.00, 70.00	98.83	2.18, 0.52	99.22
37	15-delta-prostaglandin-j2		90.00, 90.00	99.44	3.07, 0.89	99.19
38	SA-792728		80.00, 70.00	98.81	1.88, 0.68	99.19
39	7b-cis		70.00, 60.00	98.4	2.61, 0.70	99.19
40	puromycin	Protein synthesis inhibitor	90.00, 90.00	99.49	2.40, 0.51	99.15
41	AKT-inhibitor-IV		60.00, 50.00	98.09	2.85, 0.60	99.15
42	JLK-6	Gamma secretase inhibitor	70.00, 50.00	98.04	1.88, 0.56	99.11
43	BAY-11-7821	NFkB pathway inhibitor	50.00, 50.00	96.67	1.55, 0.50	99.09
44	NSC-663284		80.00, 70.00	99.04	1.94, 0.48	99.08
45	thapsigargin		55.56, 44.44	96.62	1.59, 0.48	98.94
46	triciribine		50.00, 50.00	91.8	1.44, 0.15	98.91
47	parthenolide		70.00, 50.00	98	1.69, 0.69	98.84
48	withaferin-a	IKK inhibitor	60.00, 60.00	98.45	1.85, 0.40	98.82
49	neratinib	EGFR inhibitor	50.00, 40.00	95.57	1.48, 0.31	98.8
50	LDN-193189		50.00, 50.00	95.77	4.48, 1.43	98.77
51	CA-074-Me		77.78, 77.78	98.73	2.63, 0.91	98.73
52	devazepide	CCK receptor antagonist	40.00, 40.00	93.88	1.43, 0.45	98.7
53	AG-592		100.00, 90.00	99.15	1.82, 0.48	98.66
54	15-delta-prostaglandin-j2	PPAR receptor agonist	40.00, 40.00	89.71	1.39, 0.63	98.66
55	triciribine		40.00, 40.00	88.97	1.63, 0.16	98.66
56	alvespimycin	HSP inhibitor	70.00, 60.00	98.72	1.89, 0.56	98.59
57	flavokavain-b		60.00, 60.00	98.07	1.49, 0.66	98.54
58	pyrrolidine-dithiocarbamate	NFkB pathway inhibitor	80.00, 70.00	98.61	1.64, 0.73	98.52
59	quinoclamine		90.00, 50.00	97.96	2.48, 0.58	98.48
60	BCL2-inhibitor	BCL inhibitor	50.00, 40.00	94.21	1.31, 0.45	98.45
61	IKK-2-inhibitor-V	IKK inhibitor, NFkB pathway inhibitor	100.00, 90.00	98.85	1.99, 0.72	98.31
62	CGK-733		80.00, 80.00	98.56	2.44, 0.64	98.27
63	CGP-71683		80.00, 70.00	98.34	1.72, 0.61	98.26
64	cercosporin		88.89, 88.89	98.23	2.96, 0.78	98.23
65	thapsigargin	ATPase inhibitor	60.00, 50.00	97.65	1.76, 0.47	98.21
66	SA-792541		50.00, 50.00	90.29	0.84, 0.23	98.19
67	niclosamide		80.00, 60.00	98.26	2.25, 0.74	98.13
68	azacitidine		66.67, 44.44	96.17	2.08, 0.81	98.06
69	RO-28-1675		70.00, 70.00	98.32	0.66, 0.01	98.03
70	BMY-45778		44.44, 33.33	91.63	1.58, 0.56	97.96
71	chloroxine		60.00, 60.00	97.82	1.31, 0.13	97.92
72	BIIB021	HSP inhibitor	50.00, 50.00	95.67	1.28, 0.52	97.92
73	isoliquiritigenin		80.00, 50.00	97.24	1.63, 0.59	97.88
74	CCCP		80.00, 60.00	98.09	2.15, 0.60	97.85
75	thiazolopyrimidine		40.00, 30.00	60.66	0.32, 0.08	97.82

76	tegaserod		85.71, 57.14	97.81	1.69, 0.63	97.81
77	FCCP		90.00, 60.00	97.94	1.90, 0.72	97.67
78	VU-0418947-2	HIF activator	66.67, 66.67	97.65	1.82, 0.63	97.65
79	SID-26681509		60.00, 50.00	96.78	0.13, 0.02	97.57
80	capsazepine		50.00, 40.00	94.46	1.49, 0.49	97.54
81	IKK-16	IKK inhibitor	60.00, 60.00	97.55	1.49, 0.32	97.53
82	dorsomorphin		60.00, 50.00	97.42	1.16, 0.24	97.53
83	penfluridol	T-type calcium channel blocker	90.00, 50.00	97.74	1.57, 0.35	97.46
84	menadione		40.00, 30.00	90.66	2.21, 0.41	97.46
85	heliomycin		66.67, 44.44	97.43	2.15, 0.80	97.43
86	terreic-acid		40.00, 20.00	86.38	1.56, 0.52	97.39
87	fenretinide	Retinoid receptor agonist	30.00, 20.00	87.97	0.82, 0.16	97.3
88	tyrphostin-A9		70.00, 40.00	97.15	2.16, 0.70	97.29
89	angiogenesis-inhibitor		50.00, 10.00	91.45	1.67, 0.48	97.25
90	SCH-79797		66.67, 33.33	97.13	3.38, 0.63	97.22
91	BVT-948		55.56, 33.33	95.22	2.32, 0.45	97.12
92	VU-0418946-1	HIF activator	70.00, 50.00	97.72	1.80, 0.63	97.11
93	importazole		50.00, 30.00	95.8	1.51, 0.24	97.06
94	cyclosporin-a		70.00, 40.00	97.17	2.28, 0.90	97.05
95	lylamine		50.00, 40.00	95.45	1.65, 0.63	97.03
96	cyclosporin-a		60.00, 30.00	95.92	1.56, 0.48	96.97
97	BRD-K98824517		50.00, 30.00	93.53	2.49, 0.73	96.86
98	GW-405833	Cannabinoid receptor agonist	50.00, 40.00	94.53	0.10, 0.01	96.83
99	QL-XII-47		40.00, 30.00	92.08	2.59, 0.67	96.83
100	pyrvinium-pamoate		80.00, 40.00	96.92	2.96, 0.81	96.76
101	chaetocin		30.00, 10.00	89.68	0.70, 0.05	96.72
102	avrainvillamide-analog-2	Nucleophosmin inhibitor	11.11, 0.00	77.64	0.59, 0.16	96.69
103	JAK3-inhibitor-VI	JAK inhibitor	50.00, 30.00	95.53	1.86, 0.65	96.64
104	brefeldin-a	Protein synthesis inhibitor	50.00, 30.00	94.62	2.14, 0.78	96.49
105	vinblastine	Tubulin inhibitor	50.00, 20.00	94.6	1.43, 0.39	96.49
106	calmidazolium		50.00, 37.50	95.59	2.63, 0.69	96.48
107	elesclomol		40.00, 20.00	67.24	1.14, 0.13	96.24
108	rottlerin		70.00, 50.00	96.88	2.56, 0.93	96.23
109	rhodomyrtoxin-b		77.78, 44.44	96.94	1.80, 0.58	96.19
110	calmidazolium		44.44, 22.22	93.65	2.27, 0.38	96.19
111	vinblastine		40.00, 30.00	82.22	1.72, 0.49	96.19
112	phorbol-12-myristate-13-acetate	PKC activator	40.00, 10.00	94.4	2.48, 0.49	96.13
113	LE-135		40.00, 30.00	90.94	0.53, 0.05	96.09
114	amsacrine	Topoisomerase inhibitor	50.00, 30.00	95.22	2.77, 0.68	96.03
115	triclosan		40.00, 20.00	79.76	1.36, 0.30	96
116	malonoben		80.00, 50.00	97.63	1.84, 0.60	95.99
117	CD-437		70.00, 50.00	96.99	3.42, 0.89	95.98
118	JW-7-24-1		60.00, 30.00	96.61	2.46, 0.67	95.91
119	methylene-blue		40.00, 20.00	93.83	2.94, 0.64	95.88
120	everolimus		50.00, 20.00	94.13	1.93, 0.51	95.87
121	purvalanol-a	CDK inhibitor	44.44, 33.33	93.59	2.88, 0.41	95.84
122	ivermectin		70.00, 30.00	95.74	1.89, 0.43	95.78
123	bithionol		60.00, 20.00	96.24	1.89, 0.60	95.77
124	ryuvidine		40.00, 30.00	92.22	1.86, 0.38	95.73
125	disulfiram		37.50, 25.00	87.4	1.80, 0.52	95.69
126	sappanone-a		50.00, 20.00	94.36	1.13, 0.48	95.66
127	BI-78D3		40.00, 10.00	70.61	1.74, 0.51	95.63
128	sirolimus		50.00, 20.00	91.32	0.92, 0.30	95.61
129	BAX-channel-blocker		40.00, 10.00	69.09	2.48, 0.57	95.6
130	securinine		40.00, 10.00	93.33	1.26, 0.41	95.56
131	YM-155		30.00, 20.00	92.55	2.30, 0.64	95.48
132	perhexiline		40.00, 20.00	86.2	2.63, 0.72	95.45
133	GSK-1059615	MTOR inhibitor, PI3K inhibitor	22.22, 11.11	88.07	1.64, 0.68	95.42
134	obatoclax	BCL inhibitor	60.00, 30.00	95.94	2.15, 0.70	95.38
135	cyclopiazonic-acid	ATPase inhibitor	40.00, 20.00	90.78	1.99, 0.56	95.38
136	selamectin		60.00, 20.00	95.95	1.74, 0.50	95.35
137	anisomycin		55.56, 22.22	95.24	3.61, 0.86	95.24
138	digitoxin	ATPase inhibitor	40.00, 30.00	85.71	2.18, 0.56	95.14
139	afatinib	EGFR inhibitor	50.00, 20.00	93.22	1.34, 0.32	95.11
140	NNC-55-0396	T-type calcium channel blocker	40.00, 40.00	93.4	1.65, 0.48	94.96
141	LY-2183240		20.00, 20.00	88.48	1.76, 0.63	94.91
142	narciclasine		30.00, 0.00	94.8	3.52, 0.82	94.85
143	mebendazole	Tubulin inhibitor	20.00, 0.00	84.43	1.63, 0.59	94.71
144	panobinostat	HDAC inhibitor	10.00, 0.00	90.1	1.89, 0.52	94.7
145	AG-879	EGFR inhibitor, VEGFR inhibitor	60.00, 10.00	96.39	2.11, 0.65	94.64
146	tunicamycin		66.67, 0.00	95.47	2.39, 0.75	94.64
147	vincristine	Tubulin inhibitor	12.50, 12.50	77.93	1.57, 0.50	94.63
148	clofarabine	Ribonucleotide reductase inhibitor	10.00, 10.00	92.62	2.16, 0.52	94.57
149	cephaeline	Protein synthesis inhibitor	11.11, 0.00	77.68	3.33, 0.78	94.5
150	bufalin	ATPase inhibitor	20.00, 20.00	88.25	2.18, 0.60	94.43

151	prostratin	PKC activator	10.00, 10.00	88.75	3.63, 1.03	94.4
152	NVP-AUY922	HSP inhibitor	30.00, 10.00	93.27	1.22, 0.41	94.33
153	digoxin		10.00, 0.00	90.21	2.17, 0.51	94.33
154	KI-8751	PDGFR/KIT inhibitor	22.22, 22.22	66.68	2.49, 0.72	94.29
155	mitomycin-c	DNA synthesis inhibitor	20.00, 20.00	90.06	1.81, 0.56	94.19
156	calyculin		11.11, 0.00	77.62	2.60, 0.66	94.17
157	4-hydroxy-2-nonenal		33.33, 16.67	84.56	1.76, 0.58	94.1
158	digitoxigenin	ATPase inhibitor	11.11, 11.11	92.35	2.61, 0.56	94.08
159	digoxin	ATPase inhibitor	30.00, 30.00	84.54	2.18, 0.59	94.07
160	homoharringtonine	Protein synthesis inhibitor	11.11, 11.11	92.08	2.88, 0.75	94.01
161	geldanamycin	HSP inhibitor	30.00, 20.00	93	0.99, 0.30	93.93
162	vincristine		20.00, 10.00	86.67	1.41, 0.49	93.88
163	aminopurvalanol-a	CDK inhibitor	30.00, 20.00	92.51	1.91, 0.49	93.83
164	strophanthidin	ATPase inhibitor	30.00, 20.00	81.22	1.91, 0.48	93.82
165	penicillic-acid		11.11, 0.00	90.36	1.40, 0.55	93.73
166	cinobufagin	ATPase inhibitor	20.00, 20.00	85.29	2.39, 0.55	93.69
167	HU-211		11.11, 11.11	66.43	2.10, 0.49	93.51
168	tyrphostin-AG-1478	EGFR inhibitor	40.00, 10.00	93.09	1.60, 0.51	93.49
169	BRD-K73610817		22.22, 22.22	91.65	1.99, 0.45	93.44
170	vincristine		20.00, 0.00	73.67	1.67, 0.57	93.4
171	oligomycin-a	ATP synthase inhibitor, ATPase inhibitor	20.00, 0.00	93.24	1.85, 0.44	93.38
172	niguldipine		40.00, 10.00	93.1	1.43, 0.32	93.36
173	D-64406	PDGFR/KIT inhibitor	10.00, 10.00	89.51	1.03, 0.28	93.33
174	TW-37	BCL inhibitor	30.00, 0.00	92.61	2.60, 0.72	93.32
175	PAC-1		22.22, 0.00	69.48	2.34, 0.56	93.2
176	terfenadine		30.00, 20.00	93.65	1.94, 0.60	93.14
177	apicidin	HDAC inhibitor	20.00, 0.00	84.52	1.56, 0.50	93.13
178	BRD-K37940862		22.22, 22.22	91.05	2.22, 0.74	92.92
179	periplocymarin		11.11, 0.00	90.59	2.74, 0.73	92.88
180	exemestane	Aromatase inhibitor	10.00, 0.00	90.52	1.02, 0.35	92.61
181	lasalocid		33.33, 11.11	92.6	2.03, 0.66	92.6
182	emetine	Protein synthesis inhibitor	0.00, 0.00	59.79	2.92, 0.68	92.49
183	emetine		12.50, 0.00	80.08	3.28, 0.78	92.44
184	BI-2536	Bromodomain Inhibitor	10.00, 10.00	89.56	1.49, 0.56	92.35
185	artesunate		0.00, 0.00	69.49	1.72, 0.51	92.32
186	ouabain	ATPase inhibitor	10.00, 10.00	83.18	2.09, 0.56	92.04
187	NVP-TAE684		10.00, 10.00	85.42	1.82, 0.51	92.01
188	helveticoside	ATPase inhibitor	11.11, 0.00	83.88	2.44, 0.81	91.9
189	mirin		20.00, 10.00	66.76	1.16, 0.47	91.75
190	ispinesib		20.00, 20.00	90.58	1.68, 0.51	91.72
191	RO-3306	CDK inhibitor	22.22, 22.22	68.59	1.35, 0.31	91.7
192	RS-17053		20.00, 10.00	91.63	1.68, 0.52	91.68
193	rottlerin		30.00, 20.00	69.15	1.28, 0.32	91.61
194	PD-198306	MEK inhibitor	30.00, 20.00	68.83	1.14, 0.31	91.61
195	VU-0365114-2		0.00, 0.00	60.35	1.65, 0.58	91.61
196	PHA-665752		20.00, 0.00	81.9	1.49, 0.38	91.53
197	DL-PDMP		20.00, 20.00	88.01	0.67, 0.05	91.38
198	CGS-15943		22.22, 22.22	74.16	1.56, 0.28	91.34
199	ouabain		20.00, 0.00	78.3	2.21, 0.51	91.32
200	Ala-Ala-Phe-CMK		22.22, 0.00	89.04	1.13, 0.43	91.23
201	tosyl-phenylalanyl-chloromethyl-ketone		20.00, 20.00	50.22	1.01, 0.35	91.01
202	torin-2	MTOR inhibitor	20.00, 0.00	64.39	1.56, 0.48	90.99
203	xanthohumol		10.00, 10.00	66.31	1.35, 0.38	90.92
204	III606050		0.00, 0.00	70.74	1.08, 0.27	90.78
205	WAY-170523		10.00, 10.00	91.06	1.30, 0.38	90.71
206	artesunate		12.50, 12.50	79.36	0.98, 0.23	90.68
207	cyclosporin-a		20.00, 0.00	89.52	1.84, 0.53	90.56
208	sarmentogenin	ATPase inhibitor	0.00, 0.00	86.24	2.40, 0.72	90.55
209	BX-912		0.00, 0.00	82.06	0.92, 0.26	90.45
210	doxorubicin		10.00, 0.00	70.86	1.58, 0.51	90.38
211	triciribine		10.00, 0.00	58.37	0.97, 0.24	90.29
212	oligomycin-c	ATP synthase inhibitor, ATPase inhibitor	12.50, 12.50	85.4	1.44, 0.43	90.15
213	nocodazole	Tubulin inhibitor	10.00, 0.00	61.21	1.52, 0.57	90.1
214	ingenol	PKC activator	10.00, 10.00	69.86	3.22, 0.82	90.06
215	phloretin		10.00, 0.00	77.99	0.17, 0.02	90.02

B

rank	name	belongs (PCL)	pc	median_score	ts_pc	score
			HT29			
1	SA-792728		80.00, 80.00	99.86	1.88, 0.68	99.93
2	BMY-45778		66.67, 66.67	99.67	1.58, 0.56	99.89
3	AG-957		70.00, 60.00	99.66	1.59, 0.56	99.89
4	sirolimus		60.00, 60.00	99.31	0.92, 0.30	99.89
5	caffeic-acid		70.00, 60.00	99.31	1.24, 0.43	99.75
6	tyrphostin-AG-556		50.00, 50.00	95.01	1.13, 0.40	99.75
7	lasalocid		60.00, 60.00	99.33	1.80, 0.35	99.68
8	capsazepine		60.00, 60.00	98.63	1.49, 0.49	99.68
9	cyclopiazonic-acid	ATPase inhibitor	60.00, 60.00	98.61	1.99, 0.56	99.65
10	thapsigargin		55.56, 55.56	98.54	1.59, 0.48	99.65
11	radicicol	HSP inhibitor	80.00, 80.00	99.71	2.09, 0.76	99.58
12	thapsigargin	ATPase inhibitor	60.00, 60.00	99.51	1.76, 0.47	99.58
13	WR-216174		60.00, 60.00	98.56	1.34, 0.45	99.58
14	oligomycin-c	ATP synthase inhibitor, ATPase inhibitor	62.50, 62.50	99.39	1.44, 0.43	99.54
15	piceatannol		50.00, 50.00	87.37	1.05, 0.30	99.54
16	cyclosporin-a		80.00, 80.00	99.23	1.56, 0.48	99.51
17	ABT-737	BCL inhibitor	60.00, 50.00	97.49	1.83, 0.67	99.51
18	BAY-11-7821	NFkB pathway inhibitor	50.00, 50.00	93.53	1.55, 0.50	99.51
19	suloctidil		77.78, 77.78	99.4	2.07, 0.56	99.47
20	cyclosporin-a		50.00, 50.00	96.14	1.15, 0.44	99.45
21	CGP-71683		100.00, 80.00	99.42	1.72, 0.61	99.4
22	EI-346-erlotinib-analog		70.00, 70.00	99.22	1.74, 0.51	99.37
23	BCL2-inhibitor	BCL inhibitor	50.00, 50.00	82.22	1.31, 0.45	99.36
24	NVP-AUY922	HSP inhibitor	80.00, 80.00	99.11	1.22, 0.41	99.3
25	cyclosporin-a		100.00, 90.00	99.19	1.84, 0.53	99.26
26	MG-132	Proteasome inhibitor	100.00, 90.00	99.39	2.30, 0.69	99.19
27	BCI-hydrochloride		70.00, 70.00	99.18	2.47, 0.52	99.15
28	manumycin-a	NFkB pathway inhibitor	70.00, 70.00	98.84	2.32, 0.57	99.15
29	brefeldin-a	Protein synthesis inhibitor	100.00, 90.00	99.25	2.14, 0.78	99.12
30	manumycin-a		60.00, 60.00	98.71	3.15, 0.77	99.12
31	geldanamycin	HSP inhibitor	70.00, 70.00	99.12	0.99, 0.30	99.08
32	MLN-2238	Proteasome inhibitor	90.00, 80.00	99.1	2.44, 0.85	99.08
33	z-leu3-VS	Proteasome inhibitor	100.00, 100.00	99.22	3.00, 0.82	99.04
34	NSC-632839		70.00, 60.00	98.79	3.01, 0.78	98.94
35	diphencyprone		62.50, 62.50	98.66	1.84, 0.66	98.94
36	thiostrepton	Protein synthesis inhibitor	90.00, 80.00	99.24	1.86, 0.31	98.91
37	brazilin		70.00, 40.00	96.83	1.50, 0.50	98.91
38	tyrphostin-AG-1478	EGFR inhibitor	100.00, 90.00	99.11	1.60, 0.51	98.87
39	lasalocid		77.78, 77.78	99.04	2.03, 0.66	98.86
40	bithionol		50.00, 50.00	95.51	1.89, 0.60	98.8
41	isoliquiritigenin		80.00, 80.00	99.1	1.63, 0.59	98.77
42	SA-1478088		90.00, 70.00	98.27	2.35, 0.81	98.73
43	15-delta-prostaglandin-j2	PPAR receptor agonist	50.00, 50.00	96.43	1.39, 0.63	98.73
44	GW-405833	Cannabinoid receptor agonist	60.00, 50.00	97.95	0.10, 0.01	98.7
45	butein		80.00, 50.00	97.86	1.52, 0.56	98.63
46	sappanone-a		60.00, 40.00	96.03	1.13, 0.48	98.59
47	15-delta-prostaglandin-j2		90.00, 90.00	99.02	3.07, 0.89	98.56
48	SA-792709		90.00, 80.00	98.81	1.50, 0.55	98.56
49	arachidonyl-trifluoro-methane		40.00, 40.00	81.14	1.40, 0.15	98.52
50	RO-28-1675		70.00, 60.00	98.47	0.66, 0.01	98.38
51	SID-26681509		50.00, 50.00	94.51	0.13, 0.02	98.38
52	NSC-632839		80.00, 80.00	98.71	2.25, 0.61	98.34
53	NSC-3852	HDAC inhibitor	70.00, 60.00	98.7	2.16, 0.67	98.34
54	CGK-733		90.00, 60.00	98.52	2.44, 0.64	98.34
55	CA-074-Me		77.78, 66.67	97.94	2.63, 0.91	98.34
56	rottlerin		70.00, 50.00	97.28	2.56, 0.93	98.25
57	SSR-69071		70.00, 50.00	97.52	2.18, 0.52	98.19
58	kinetin-riboside		70.00, 60.00	98.17	2.90, 0.75	98.13
59	PD-198306	MEK inhibitor	50.00, 50.00	92.49	1.14, 0.31	98.06
60	parthenolide	NFkB pathway inhibitor	70.00, 60.00	98.12	2.27, 0.65	97.99
61	rottlerin		50.00, 40.00	94.72	1.28, 0.32	97.93
62	cyclosporin-a		100.00, 90.00	98.88	2.28, 0.90	97.92
63	tunicamycin		77.78, 77.78	98.59	2.39, 0.75	97.92
64	flavokavain-b		60.00, 50.00	96.54	1.49, 0.66	97.85
65	malonoben		80.00, 60.00	97.94	1.84, 0.60	97.83
66	FCCP		50.00, 50.00	96.08	1.90, 0.72	97.78
67	tegaserod		85.71, 71.43	97.94	1.69, 0.63	97.74
68	AG-592		90.00, 80.00	97.83	1.82, 0.48	97.67
69	exemestane	Aromatase inhibitor	50.00, 20.00	94.9	1.02, 0.35	97.67
70	niguldipine		70.00, 40.00	97.05	1.43, 0.32	97.66
71	DL-PDMP		70.00, 40.00	97.03	0.67, 0.05	97.65
72	spiperone		30.00, 30.00	45.6	0.64, 0.16	97.65
73	puromycin		80.00, 70.00	98.19	2.26, 0.58	97.6
74	lylamine		50.00, 40.00	94.86	1.65, 0.63	97.6
75	pyrrolidine-dithiocarbamate	NFkB pathway inhibitor	70.00, 30.00	96.26	1.64, 0.73	97.57
76	cucurbitacin-i		70.00, 40.00	97.15	2.92, 0.80	97.32
77	disulfiram		62.50, 25.00	96.23	1.80, 0.52	97.31
78	fenretinide	Retinoid receptor agonist	50.00, 30.00	86.43	0.82, 0.16	97.31
79	iodoacetic-acid		44.44, 33.33	80.92	1.67, 0.34	97.16
80	IKK-2-inhibitor-V	IKK inhibitor, NFkB pathway inhibitor	70.00, 60.00	97.78	1.99, 0.72	96.95
81	piperlongumine		60.00, 40.00	96.17	2.11, 0.70	96.93

82	alvespimycin	HSP inhibitor	40.00, 20.00	93.99	1.89, 0.56	96.9
83	cucurbitacin-i		90.00, 70.00	98.31	2.09, 0.45	96.85
84	SA-792987	PKC inhibitor	50.00, 20.00	83.78	1.32, 0.30	96.82
85	BRD-K48974000		55.56, 22.22	96.1	0.70, 0.22	96.81
86	penfluridol	T-type calcium channel blocker	70.00, 50.00	97.47	1.57, 0.35	96.74
87	7b-cis		60.00, 40.00	96.16	2.61, 0.70	96.72
88	niclosamide		70.00, 20.00	96.88	2.25, 0.74	96.44
89	JLK-6	Gamma secretase inhibitor	50.00, 30.00	94.02	1.88, 0.56	96.44
90	WAY-170523		50.00, 20.00	94.58	1.30, 0.38	96.41
91	phenethyl-isothiocyanate		30.00, 10.00	91.69	0.93, 0.19	96.34
92	rhodomyrtoxin-b		55.56, 44.44	96.33	1.80, 0.58	96.33
93	indirubin	Glycogen synthase kinase inhibitor	44.44, 22.22	93.46	1.80, 0.53	96.3
94	pifithrin-mu		40.00, 10.00	84.14	1.05, 0.24	96.3
95	SCH-79797		44.44, 22.22	94.21	3.38, 0.63	96.23
96	withaferin-a	IKK inhibitor	40.00, 10.00	91.22	1.85, 0.40	96.19
97	securinine		30.00, 0.00	89.25	1.26, 0.41	96.12
98	JTC-801		66.67, 16.67	95.39	2.92, 0.63	96.09
99	devazepide	CCK receptor antagonist	30.00, 20.00	79.57	1.43, 0.45	95.95
100	selamectin		50.00, 30.00	92.53	1.74, 0.50	95.91
101	CCCP		70.00, 30.00	95.87	2.15, 0.60	95.88
102	terfenadine		50.00, 30.00	93.4	1.94, 0.60	95.87
103	parthenolide		40.00, 10.00	89.2	1.69, 0.69	95.82
104	IKK-16	IKK inhibitor	40.00, 10.00	86.54	1.49, 0.32	95.78
105	chloroxine		40.00, 30.00	92.06	1.31, 0.13	95.77
106	ZK-164015	Estrogen receptor antagonist	40.00, 10.00	92.52	1.38, 0.18	95.7
107	BNTX		55.56, 33.33	95.11	2.36, 0.59	95.66
108	NNC-55-0396	T-type calcium channel blocker	50.00, 30.00	89.46	1.65, 0.48	95.66
109	LE-135		40.00, 30.00	89.9	0.53, 0.05	95.53
110	calmidazolium		44.44, 22.22	94.8	2.27, 0.38	95.46
111	quinoclamine		70.00, 30.00	95.74	2.48, 0.58	95.38
112	NSC-663284		50.00, 20.00	93.79	1.94, 0.48	95.28
113	GSK-3-inhibitor-IX	Glycogen synthase kinase inhibitor, PKC inhibitor	30.00, 10.00	91.07	1.55, 0.44	95.24
114	azacitidine		33.33, 22.22	89.93	2.08, 0.81	95.18
115	puromycin	Protein synthesis inhibitor	80.00, 30.00	96.52	2.40, 0.51	95.14
116	T-98475		60.00, 30.00	95.82	1.59, 0.49	95.14
117	PK-11195		30.00, 20.00	56.13	0.45, 0.03	95.1
118	AKT-inhibitor-IV		50.00, 20.00	89.92	2.85, 0.60	95
119	penitrem-a		30.00, 30.00	91.05	0.89, 0.24	94.95
120	vinblastine		30.00, 20.00	85.08	1.72, 0.49	94.94
121	mefloquine		22.22, 11.11	90.69	1.34, 0.28	94.88
122	ivermectin		30.00, 10.00	93.69	1.89, 0.43	94.68
123	Y-134	Estrogen receptor antagonist	30.00, 30.00	83.06	1.28, 0.23	94.61
124	CD-437		30.00, 30.00	77.72	3.42, 0.89	94.61
125	pyrvinium-pamoate		30.00, 10.00	92.53	2.96, 0.81	94.32
126	5-nonyloxytryptamine		40.00, 10.00	91.49	1.58, 0.38	94.21
127	tyrphostin-A9		40.00, 20.00	89.16	2.16, 0.70	94.15
128	angiogenesis-inhibitor		20.00, 10.00	88.54	1.67, 0.48	93.97
129	neratinib	EGFR inhibitor	30.00, 30.00	82.98	1.48, 0.31	93.89
130	AG-879	EGFR inhibitor, VEGFR inhibitor	40.00, 20.00	92.94	2.11, 0.65	93.76
131	cercosporin		55.56, 33.33	95.29	2.96, 0.78	93.73
132	niguldipine		50.00, 30.00	95.12	2.22, 0.72	93.73
133	perhexiline		30.00, 30.00	59.48	2.63, 0.72	93.55
134	auranofin	NFkB pathway inhibitor	30.00, 20.00	43.59	2.74, 0.50	93.49
135	ispinesib		30.00, 0.00	88.41	1.68, 0.51	93.45
136	triciribine		20.00, 10.00	85.35	1.44, 0.15	93.34
137	BRD-K37940862		22.22, 11.11	93.33	2.22, 0.74	93.33
138	JTE-907	Cannabinoid receptor agonist	30.00, 10.00	79.49	1.17, 0.34	93.32
139	BRD-K98824517		30.00, 10.00	89.86	2.49, 0.73	93.3
140	guggulsterone		20.00, 20.00	86.94	0.94, 0.31	93.29
141	BRD-K91781484		0.00, 0.00	91.05	1.51, 0.56	93.22
142	RS-17053		30.00, 20.00	90.7	1.68, 0.52	93.2
143	reserpine		20.00, 10.00	85.83	1.59, 0.30	92.99
144	SA-792541		0.00, 0.00	79.14	0.84, 0.23	92.92
145	purvalanol-a	CDK inhibitor	22.22, 22.22	65.79	2.88, 0.41	92.85
146	menadione		20.00, 20.00	72.6	2.21, 0.41	92.69
147	BVT-948		22.22, 11.11	60.97	2.32, 0.45	92.69
148	vinblastine	Tubulin inhibitor	20.00, 10.00	79.7	1.43, 0.39	92.35
149	FK-888	Tachykinin antagonist	10.00, 10.00	87.64	1.00, 0.35	92.32
150	eriodictyol		30.00, 20.00	71.54	1.48, 0.51	92.16
151	avrainvillamide-analog-2	Nucleophosmin inhibitor	11.11, 0.00	65.92	0.59, 0.16	92.15
152	MLN-4924		20.00, 0.00	85.09	2.53, 0.74	92.07
153	sirolimus	MTOR inhibitor	33.33, 11.11	69.91	1.99, 0.56	92
154	TPCA-1	IKK inhibitor	20.00, 0.00	81.91	1.81, 0.59	91.91
155	heliomycin		33.33, 0.00	91.9	2.15, 0.80	91.9
156	CMPD-1		22.22, 22.22	85.75	2.41, 0.70	91.86
157	elesclomol		10.00, 10.00	72.26	1.14, 0.13	91.83
158	importazole		20.00, 10.00	85.65	1.51, 0.24	91.65
159	penicillic-acid		22.22, 0.00	89.03	1.40, 0.55	91.31
160	thiazolopyrimidine		20.00, 20.00	39.48	0.32, 0.08	91.08
161	tyrphostin-AG-527		11.11, 11.11	81.52	1.23, 0.41	90.58
162	pimozide	Dopamine receptor antagonist	10.00, 0.00	88.2	1.60, 0.57	90.56

C

HepG2					HT29				
rank	PCL name	pc	median_score	score	rank	PCL name	pc	median_score	score
1	Proteasome inhibitor	100.00, 100.00	99.87	99.94	1	Vesicular Transport LOF	80.00, 70.00	99.17	99.87
2	NFkB pathway inhibitor	100.00, 100.00	99.9	99.9	2	Proteasome inhibitor	100.00, 90.00	99.6	99.64
3	Proteasome Pathway LOF	60.00, 60.00	98.97	99.77	3	Wnt family GOF	30.00, 30.00	80.86	99.61
4	Heat shock 70kDa proteins LOF	42.86, 42.86	93.19	99.45	4	HSP inhibitor	70.00, 70.00	99.42	99.6
5	IKK inhibitor	80.00, 70.00	99.04	99.39	5	NFkB pathway inhibitor	70.00, 70.00	99.39	99.58
6	Vesicular Transport LOF	70.00, 60.00	97.63	99.36	6	Proteasome Pathway LOF	60.00, 40.00	96.1	99.56
7	HSP inhibitor	80.00, 70.00	98.53	99.22	7	ATP synthase inhibitor	33.33, 33.33	91.09	98.92
8	HIF activator	70.00, 50.00	97.71	99.17	8	T-type calcium channel blocker	40.00, 20.00	89.29	98.56
9	BCL inhibitor	70.00, 30.00	96.52	98.79	9	IKK inhibitor	50.00, 20.00	95.61	98.41
10	Protein synthesis inhibitor	60.00, 50.00	97.13	98.58	10	BCL inhibitor	60.00, 20.00	95.85	98.36
11	T-type calcium channel blocker	50.00, 20.00	94.84	98.38	11	Protein synthesis inhibitor	40.00, 10.00	91.5	95.55
12	PKC activator	30.00, 10.00	91.32	96.89	12	Estrogen receptor antagonist	10.00, 0.00	71.2	94.83
13	ATP synthase inhibitor	33.33, 0.00	83.84	96.76	13	HIF activator	10.00, 10.00	91.1	93.86
14	ATPase inhibitor	40.00, 30.00	88.38	95.7	14	C2 domain containing LOF	25.00, 25.00	88.17	93.47
15	Cell Cycle Inhibition GOF	10.00, 10.00	86.25	92.85	15	PKC inhibitor	20.00, 0.00	71.91	91.84
16	C2 domain containing LOF	12.50, 0.00	85.74	92.51	16	EGFR inhibitor	0.00, 0.00	85.57	90.06
17	Ubiquitin-specific peptidases LOF	10.00, 0.00	69.19	90.89					
18	EGFR inhibitor	10.00, 10.00	79.63	90.26					

Figure S3. The output data of compounds (CP) and PCL were analyzed from CLUE (score ≥ 90). CLUE treated thousands of compounds in several cells, including PC3, VCAP, A375, A549, HA1E, HCC515, HT29, MCF7, and HEPG2, to detect their gene expression profiles. The summary is reflected by these cells' connectivity scores, and thus ranking was dependent on this summary score. The abbreviation **pc** denotes the percentage of total perturbagens that queried the column sample against the Touchstone data set and exceeded the given thresholds; **ts_pc** denotes the percentage of total Touchstone perturbagens that connected to the given perturbagen above the indicated thresholds, and **median_score** denotes the average connectivity score for nine types of cells. (**A**) The gene-expression profile from HepG2 treated with curcumin was analyzed by CLUE. (**B**) The gene-expression profile from HT29 treated with curcumin was analyzed by CLUE focused on the compounds

with connectivity scores greater than 90. Because the raw data were extensive, we present only the 30 highest-scoring compounds. (**C**) More than 90 PCL scores had similar effects to those of HepG2 and HT29 treated with curcumin.

HepG2 **HT29**

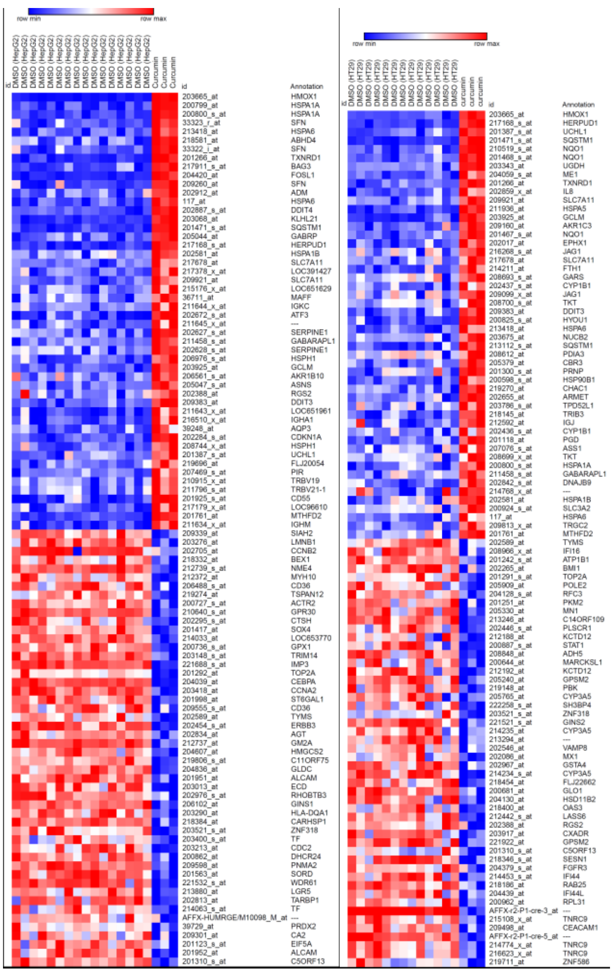

Figure S4. The heat maps for HepG2 and HT29. The heat map shows the top 50 up and down probes. Differentially expressed probe sets were selected by arbitrary (fold change) ≥ 1.5 and p-value < 0.01 (two sample t-test). Corresponding gene names of probe sets were based on HG_U133A.chip file download from GSEA website.

References

1. Lamb, J.; Crawford, E.D.; Peck, D.; Modell, J.W.; Blat, I.C.; Wrobel, M.J.; Lerner, J.; Brunet, J.P.; Subramanian, A.; Ross, K.N.; Reich, M.; Hieronymus, H.; Wei, G.; Armstrong, S.A.; Haggarty, S.J.; Clemons, P.A.; Wei, R.; Carr, S.A.; Lander, E.S.; Golub, T.R., The Connectivity Map: using gene-expression signatures to connect small molecules, genes, and disease. *Science (New York, N.Y.)* **2006,** *313*, 1929-1935.

2. Subramanian, A.; Narayan, R.; Corsello, S.M.; Peck, D.D.; Natoli, T.E.; Lu, X.; Gould, J.; Davis, J.F.; Tubelli, A.A.; Asiedu, J.K.; Lahr, D.L.; Hirschman, J.E.; Liu, Z.; Donahue, M.; Julian, B.; Khan, M.; Wadden, D.; Smith, I.C.; Lam, D.; Liberzon, A.; Toder, C.; Bagul, M.; Orzechowski, M.; Enache, O.M.; Piccioni, F.; Johnson, S.A.; Lyons, N.J.; Berger, A.H.; Shamji, A.F.; Brooks, A.N.; Vrcic, A.; Flynn, C.; Rosains, J.; Takeda, D.Y.; Hu, R.; Davison, D.; Lamb, J.; Ardlie, K.; Hogstrom, L.; Greenside, P.; Gray, N.S.; Clemons, P.A.; Silver, S.; Wu, X.; Zhao, W.N.; Read-Button, W.; Haggarty, S.J.; Ronco, L.V.; Boehm, J.S.; Schreiber, S.L.; Doench, J.G.; Bittker, J.A.; Root, D.E.; Wong, B.; Golub, T.R., A Next Generation Connectivity Map: L1000 Platform and the First 1,000,000 Profiles. *Cell* **2017,** *171*, 1437-1452 e1417.
